# Conduction Properties Distinguish Unmyelinated Sympathetic Efferent Fibers and Unmyelinated Primary Afferent Fibers in the Monkey

**DOI:** 10.1371/journal.pone.0009076

**Published:** 2010-02-05

**Authors:** Matthias Ringkamp, Lisa M. Johanek, Jasenka Borzan, Timothy V. Hartke, Gang Wu, Esther M. Pogatzki-Zahn, James N. Campbell, Beom Shim, Raf J. Schepers, Richard A. Meyer

**Affiliations:** 1 Department of Neurosurgery, School of Medicine, Johns Hopkins University, Baltimore, Maryland, United States of America; 2 Department of Anesthesiology, School of Medicine, Johns Hopkins University, Baltimore, Maryland, United States of America; 3 Applied Physics Laboratory, Johns Hopkins University, Baltimore, Maryland, United States of America; Emory University, United States of America

## Abstract

**Background:**

Different classes of unmyelinated nerve fibers appear to exhibit distinct conductive properties. We sought a criterion based on conduction properties for distinguishing sympathetic efferents and unmyelinated, primary afferents in peripheral nerves.

**Methodology/Principal Findings:**

In anesthetized monkey, centrifugal or centripetal recordings were made from single unmyelinated nerve fibers in the peroneal or sural nerve, and electrical stimuli were applied to either the sciatic nerve or the cutaneous nerve endings, respectively. In centrifugal recordings, electrical stimulation at the sympathetic chain and dorsal root was used to determine the fiber's origin. In centrifugal recordings, sympathetic fibers exhibited absolute speeding of conduction to a single pair of electrical stimuli separated by 50 ms; the second action potential was conducted faster (0.61 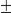 0.16%) than the first unconditioned action potential. This was never observed in primary afferents. Following 2 Hz stimulation (3 min), activity-dependent slowing of conduction in the sympathetics (8.6 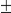 0.5%) was greater than in one afferent group (6.7 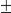 0.5%) but substantially less than in a second afferent group (29.4 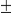 1.9%). In centripetal recordings, most mechanically-insensitive fibers also exhibited absolute speeding to twin pulse stimulation. The subset that did not show this absolute speeding was responsive to chemical stimuli (histamine, capsaicin) and likely consists of mechanically-insensitive afferents. During repetitive twin pulse stimulation, mechanosensitive afferents developed speeding, and speeding in sympathetic fibers increased.

**Conclusions/Significance:**

The presence of absolute speeding provides a criterion by which sympathetic efferents can be differentiated from primary afferents. The differences in conduction properties between sympathetics and afferents likely reflect differential expression of voltage-sensitive ion channels.

## Introduction

Unmyelinated fibers in peripheral nerves can be broadly divided into two major groups, sympathetic efferent and primary afferent fibers. Primary afferent fibers have been divided further into different subgroups based on their responsiveness to mechanical, thermal and chemical stimuli. For example, some unmyelinated afferents exclusively respond to non-noxious mechanical [1] or warm stimuli [2]. Nociceptive unmyelinated afferents have been subdivided into those that are mechanosensitive or mechanoinsensitive. Some unmyelinated mechanoinsensitive nociceptive afferents are chemonociceptors and show lasting excitation following administration of capsaicin [3] or histamine [4], [5].

The different functional classes of unmyelinated nerve fibers also differ in their conductive properties [6], [7], [8], [9]. During repetitive electrical stimulation (2 Hz, 3 min), mechanosensitive and -insensitive nociceptive afferents exhibit marked increases (>20%) in conduction latency (‘slowing’ of conduction), whereas sympathetic efferents and ‘cold’ fibers slow less (<10%)[10], [11]. These differences in activity-dependent conduction slowing are likely due to differences in expression of sodium channels in the axonal membrane [12], [13].

Immediately following an action potential (i.e., within 500 ms), the axonal membrane of unmyelinated nerve fibers undergoes time dependent excitability changes [14]. The refractory period may be followed by a phase of hyperexcitability which then is succeeded by a period of hypoexcitability. Similar to activity dependent slowing, post excitatory excitability changes appear to differ between classes of unmyelinated nerve fibers [9], [11].

In peripheral nerve recordings of cutaneous terminals, it is difficult to distinguish mechanoinsensitive afferents from sympathetic efferents since neither respond to mechanical stimuli. In human microneurography, sympathetic maneuvers (e.g., valsalva) can be used to induce a response in some sympathetic fibers, but such procedures do not distinguish afferent and efferent fibers in preparations in which axons are cut and neuronal activity is recorded from the peripheral axonal process distal to the cut. Moreover, the adequate stimulus by which mechanoinsensitive afferents could be activated is often unknown, and fibers are classified as ‘sympathetic’ by a lack of response to thermal or chemical stimuli. We therefore sought a criterion based on conductive properties that could be used to differentiate sympathetic from afferent fibers. Using centrifugal recordings in which sympathetics and afferents could be positively identified by stimulation of the sympathetic chain or the dorsal root, respectively, we found that sympathetic efferent fibers exhibited an absolute speeding of conduction to a single pair of electrical stimuli separated by 50 ms; this was never observed in primary afferent fibers. This presence of absolute speeding provides a criterion by which sympathetic efferents can be differentiated from primary afferents.

## Methods

### Ethics Statement

Experiments were approved by the Animal Care and Use Committee of the Johns Hopkins University (protocol PR06M396). Experiments were performed in accordance with Animal Welfare Act regulations and the USPHS Policy on Humane Care and Use of Laboratory Animals.

### General Procedures

Monkeys (Macaca fascicularis, 4–6 kg) were initially sedated by intramuscular ketamine (12 mg/kg with 0.04 mg/kg atropine) and anesthesia was induced by an intravenous bolus injection of sodium pentobarbital (6 mg/kg) and maintained by constant infusion (4–6 mg/kg/hr) thereof. Hydration was maintained with a continuous infusion of 5% dextrose in physiological saline solution. An intramuscular injection of penicillin (600,000 U) was given for prophylaxis against infection. Intubation was performed, and animals were paralyzed with an intravenous dose of pancuronium bromide (0.1 mg/kg) every 2 hours or when necessary. Ventilation was adjusted throughout the experiment to maintain the expired pCO2 within physiological range (i.e., 35–45 mmHg). Heart rate was monitored by an electrocardiogram to ensure an adequate level of anesthesia. Supplemental doses of pentobarbital were administered if the heart rate increased by more than 10% upon application of noxious stimuli. Core temperature was monitored via a rectal thermometer and maintained near 38°C with feedback-controlled circulating water heating pads.

Teased-fiber recording techniques [15] were used to record from single C fibers in peripheral nerves. Briefly, small filaments were cut from the nerve and placed on a dissecting platform. A smaller strand was dissected from the filament and looped around a silver-wire recording electrode. A nerve-trunk electrode was used to electrically stimulate the nerve to reveal the fibers on the recording electrode.

In experiments on 5 animals, a laminectomy over the lumbar spine was performed to gain access to the lumbar dorsal roots. After exposure of the lumbar spinal cord, the skin of the back was sutured to a metal ring to form a pool that was filled with paraffin oil. The dura was then slit open along the length of the exposed spine, and multiple dorsal roots were carefully isolated for electrical stimulation. Electrical stimulation of the dorsal roots was performed using bipolar stimulation electrodes. The anatomical level of the stimulated dorsal roots was not determined; however, stimulation of multiple roots always induced centrifugal activity that could be recorded in the superficial peroneal nerve.

In separate experiments in 4 other animals, the lumbar sympathetic chain was exposed through a retroperitoneal, dorso- lateral approach as has been described previously in rat [16]. The ramus communicans between neighboring sympathetic ganglia was positioned on a bipolar stimulation electrode allowing stimulation of the sympathetic chain. The exact anatomical level of the sympathetic ganglia at which stimulation was performed was not determined.

Animals undergoing laminectomy or exposure of the lumbar sympathetic chain were sacrificed at the end of the recordings by an overdose of pentobarbital.

### Centrifugal Recordings

**Figure 1:**
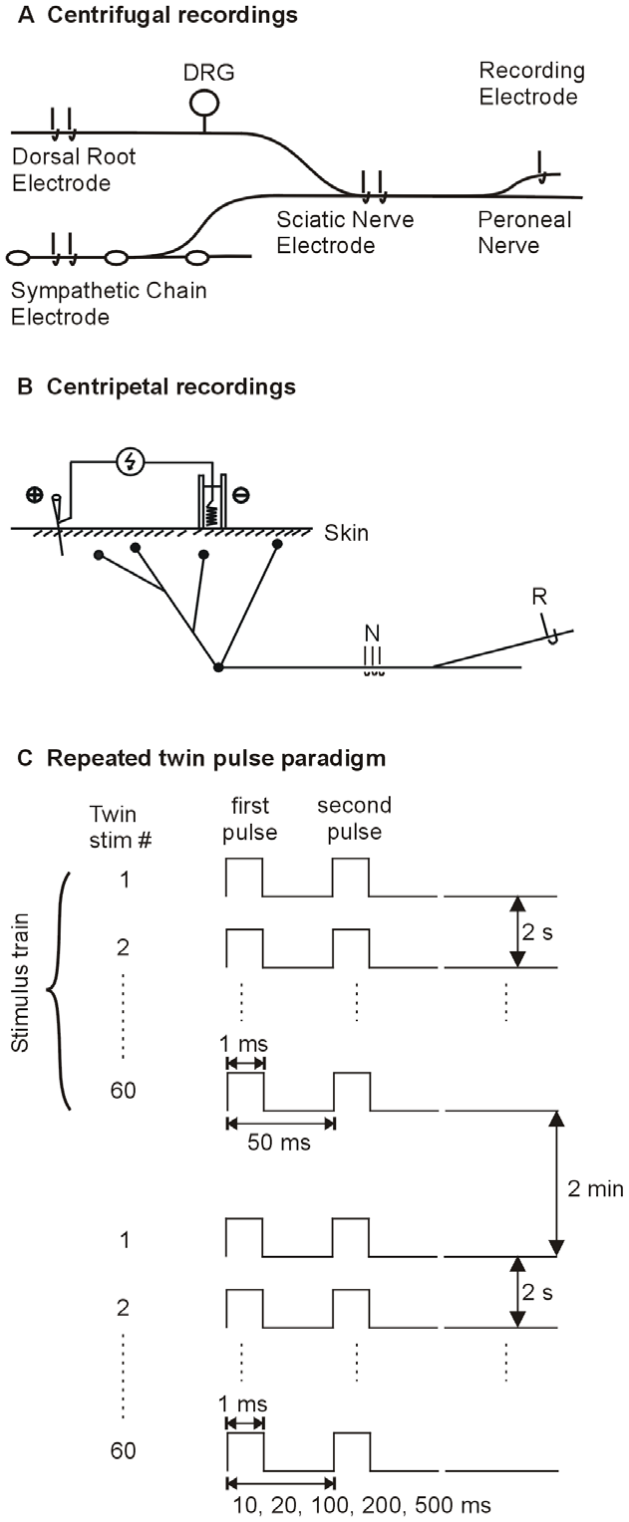
Schematic of electrophysiological preparation. **A) Teased fiber techniques were used to record action potential activity in single C fibers in the peroneal nerve.** The latencies of centrifugally propagating action potentials were obtained following electrical stimulation of the sciatic nerve. Collision techniques with stimulation at the sympathetic chain and the sciatic nerve or the dorsal root and the sciatic nerve were used to identify the peroneal nerve fiber as a sympathetic efferent fiber or a sensory afferent fiber. Only sciatic nerve fibers that were positively identified as efferent or afferent were studied. **B) Schematic drawing of the transcutaneous electrical stimulation set up used in the centripetal recordings.** Plastic wells were glued onto the skin over the receptive field of the unit under study and filled with electrode gel. A silver wire was inserted into the gel and connected to an electrical stimulator. A needle inserted outside the receptive field but nearby served as a return electrode. In some cases, the receptive field was stimulated with two ball electrodes (spacing  = 1 cm). In addition to the skin electrode, an electrode was also placed on the nerve trunk (N) between the peripheral receptive field and the recording electrode (R). **C) A repeated twin-pulse stimulation protocol was used in the centripetal recording experiments.** The first stimulus train consisted of 60 twin pulse stimuli; 50 ms separated the first and second pulse, and there was a 2 s interval between twin pulses. After a 2 min pause, additional twin pulse trains were delivered in which the interpulse interval was 10, 20, 100, 200, or 500 ms.

A schematic of the electrophysiological preparation is shown in Fig. 1A. Action potential activity was recorded in single C fibers in a cutaneous nerve (i.e, the superficial peroneal or sural nerve). The latencies of centrifugally propagating action potentials were obtained following electrical stimulation of the sciatic nerve. Bi-polar stimulation electrodes were also placed on the dorsal root or, in separate experiments, the sympathetic chain. Collision techniques with stimulation at the sympathetic chain and the sciatic nerve or the dorsal root and the sciatic nerve were used to identify the peroneal nerve fiber as a sympathetic efferent fiber or a sensory afferent fiber (see below). Only unmyelinated sciatic nerve fibers that were positively identified as efferent or afferent were studied.

The conduction distance was measured along the course of the nerve from the recording electrode to the sciatic nerve electrode. The conduction velocity was calculated by dividing this distance by the conduction latency obtained from the sciatic nerve electrode with intensities at least 1.5 times threshold for excitation. Fibers with a conduction velocity of <1.5 m/s were regarded as C fibers. Conduction velocities were also determined from the latencies following stimulation of the dorsal root or sympathetic chain electrode.

#### Collision technique to identify positively afferent and sympathetic efferent fibers

The current intensity of the electrical stimulus (1/4 Hz) at the dorsal root (or sympathetic chain) was increased until an action potential waveform at a fixed latency was observed at the peroneal nerve recording electrode. Then, a simultaneous stimulus was applied to the sciatic nerve electrode, and the current strength increased until the action potential waveform initiated from the dorsal root (or sympathetic chain) disappeared. At this current, an action potential with a waveform identical to that initiated from the dorsal root (or sympathetic chain) always appeared at a shorter latency. This ‘short latency’ action potential had been initiated at the sciatic nerve stimulation electrode and had propagated both centrifugally towards the recording electrode and centripetally towards the dorsal root (or sympathetic chain). The centripetally propagating action potential collided with the centrifugally propagating action potential initiated at the dorsal root (or sympathetic chain) thereby preventing this action potential from being conducted towards the recording electrode. This ‘collision’ provided positive evidence that an action potential waveform initiated from the sciatic nerve stimulus belonged to either a dorsal root afferent or a sympathetic efferent fiber.

#### Experimental protocol

Following a stimulus free interval of at least 2 min, two protocols for the electrical stimulation of the sciatic nerve were used in experiments in which centrifugally conducted action potentials were recorded. Stimulus strength was set at 50% above threshold for activating the C fiber of interest. 1) Twin pulse conduction: A twin pulse stimulus at an interpulse interval of 50–70 ms was used to assess whether the conduction latency of the second action potential was shorter than the first (i.e., supernormal conduction or “speeding”). 2) Activity-dependent slowing: A train of pulses (2 Hz for 3 min) was employed to investigate activity-dependent slowing of conduction. A similar protocol has been used by others to distinguish sympathetic efferent fibers from primary afferent fibers [17].

### Centripetal Recordings

In a separate series of 77 experiments in 21 animals, the conductive properties of fibers with peripheral, cutaneous terminals that were either mechanically-sensitive nociceptive afferent fibers or mechanically-insensitive fibers (efferent or afferent) were investigated. The receptive field of mechanically sensitive afferent fibers was mapped with von Frey probes. The electrical receptive field of mechanically-insensitive fibers was mapped using electrocutaneous stimulation. The electrical receptive field was defined as that region on the skin where the conduction latency decreased in discrete steps as the stimulus current increased [18].

In experiments where repeated twin pulse stimuli were delivered to the receptive field (see below), a 3-mm-diameter plastic well was glued to the skin in the receptive field and filled with electrolyte gel [19] to form a secure stimulating electrode (Fig. 1B). A coiled silver wire, positioned inside the well, served as the cathode. A stainless steel needle was inserted in the skin distal to the surface electrode and outside the receptive field. This electrode served as the anode. As the current was increased at this receptive field electrode, the latency of the recorded action potential decreased in discrete steps corresponding to excitation of the branching terminal structure at more proximal locations [19]. To insure that changes in action potential latency during our experimental protocol were not the result of hopping between different initiation sites in the peripheral terminal, we determined the largest current range over which a fixed latency was observed and set the stimulus current in the middle of this range (Fig. S1).

#### Experimental protocol

All fibers were tested with at least one twin-pulse electrical stimulus (usually with an interstimulus interval of 50 ms). In a subset of fibers, electrical test stimuli consisted of a train of 60 twin stimuli with twin stimuli applied every 2 seconds. In a given train of twin stimuli, pulses of a twin stimulus were separated by a fixed interstimulus interval (ISI) (10, 20, 50, 100, 200 or 500 ms). Trains of twin stimuli were separated by a stimulus free interval of at least 2 minutes. The stimulation protocol was started with twin pulses separated by 50 ms. The order of the remaining trains was randomized. A schematic of the stimulus protocol is presented in Fig. 1C.

### Data Collection and Analysis

A personal computer with a computer-based data acquisition board and customized data acquisition and analysis software (DAPSYS; Brian Turnquist; see www.dapsys.net) was used to display, record, and store action potential activity. On- and off-line action potential discrimination could be performed using DAPSYS. The electrical stimulators were triggered by signals from the DAPSYS software. Recorded action potentials and trigger pulses were time stamped in DAPSYS to allow for direct correlation between the two.

Data are presented as mean 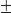 S.E.M. Data were normalized to combine latency changes across fibers: a percent change in latency was computed by dividing the latency difference by the latency of the first action potential in the stimulus protocol. Student's t-tests were used to compare data (except where noted otherwise). Sigma Plot was used to determine the linear and exponential regression lines and to determine significance of linear correlations.

## Results

A total of 48 C-fibers in the superficial peroneal nerve that were activated by electrical stimulation of the sciatic nerve were positively identified as originating from the dorsal root using collision techniques; these afferent fibers had identical mean conduction velocities when measured from the dorsal root (1.04 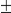 0.03 m/s) or from the sciatic nerve(1.04 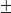 0.03 m/s) electrode. Similarly, 24 C-fibers in the superficial peroneal nerve were positively identified as originating from the sympathetic chain; these sympathetic efferent fibers had similar mean conduction velocities when measured from the sympathetic chain (1.02 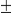 0.09 m/s) and from the sciatic nerve (0.96 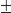 0.05 m/s) electrode.

A total of 82 C-fibers was investigated in the centripetal experiments in which the cutaneous terminal was electrically stimulated. Of these, 46 were responsive to mechanical stimuli (von Frey threshold  = 2.4 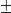 0.2 bar) and had a mean conduction velocity of 0.95 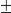 0.02 m/s. The remaining 36 fibers were insensitive to mechanical stimuli and had a mean conduction velocity of 0.87 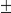 0.04 m/s.

### Sympathetic Efferent Fibers Exhibit Supra-Normal Conduction

**Figure 2:**
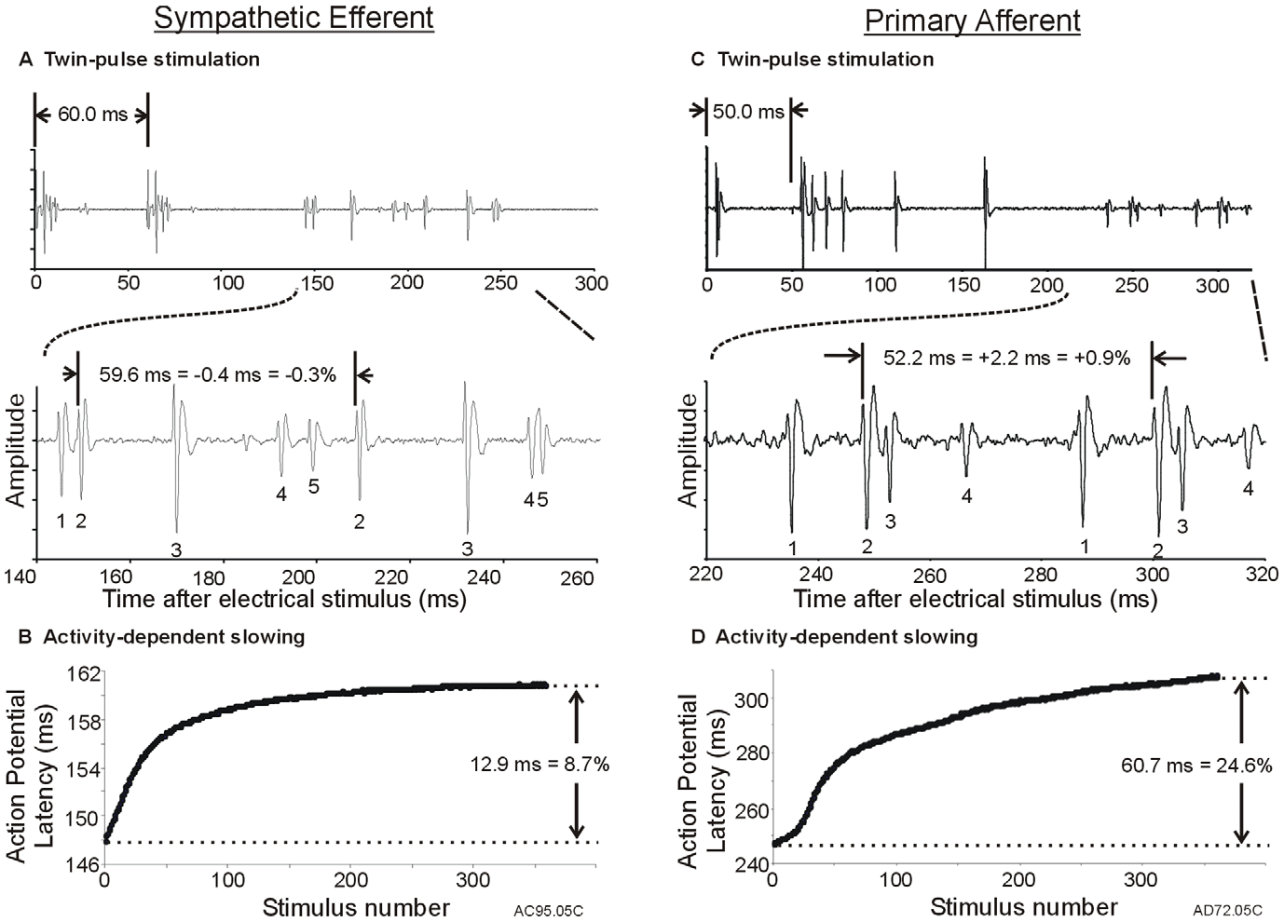
Typical results from a sympathetic efferent and a primary afferent. **A) Sympathetic efferent fiber exhibiting speeding of conduction to twin-pulse stimulation.** Following an electrical twin pulse at the sciatic nerve (60 ms interstimulus interval), the conduction latency of the second action potential (AP) for fiber #2 was 0.4 ms smaller than for the first AP. Thus, the conduction velocity of the second AP increased (i.e., “speeding”). Top: Neuronal activity (total sweep length 300 ms) with stimulus artifacts at 60 ms. Bottom: Expanded view of neuronal activity showing five different AP waveforms. Fiber #2 originated from a sympathetic efferent (as identified by collision following electrical stimulation at the sympathetic chain). The other C fibers were not positively identified. **B) Sympathetic efferent fiber shows little activity-dependent slowing.** Following a train of electrical stimuli (2 Hz, 3 min), the conduction latency for fiber #2 increased by 12.9 ms or 8.7% relative to the latency for the first AP (148.6 ms, conduction velocity  = 1.0 m/s). **C) The primary afferent fiber exhibited slowing of conduction to twin-pulse stimulation.** Following electrical twin pulses at the sciatic nerve (50 ms interstimulus interval) the conduction latency of the second AP was 2.2 ms greater than that of the first. Thus, the conduction velocity of the second AP decreased (i.e., “slowing”). Top: Neuronal activity (total sweep length: 400 ms) showing stimulus artifact at 50 ms. Bottom: Expanded view of neuronal activity showing four different AP waveforms. Fibers #1–3 were all primary afferent fibers (as identified by collision following stimulation from the electrode on the dorsal root). **D) The primary afferent fiber showed pronounced activity-dependent slowing.** Following a train of electrical pulses applied at the sciatic nerve (2 Hz, 3 min), the conduction latency for fiber #2 increased by 60.7 ms or 24.6% relative to the latency for first AP (247.6 ms, conduction velocity  = 0.93 m/s).

In response to two electrical pulses (inter pulse interval 50 to 70 ms) delivered at the sciatic nerve electrode, most of the sympathetic efferent fibers exhibited a speeding of conduction; the second action potential was conducted at a faster conduction velocity than the first action potential. For the example shown in Fig. 2A, the interstimulus interval was 60 ms. The action potential at a latency of 148.6 ms (waveform # 2) was positively identified as belonging to a sympathetic efferent based on collision experiments (see methods). The second action potential from this fiber arrived at the recording electrode 59.6 ms after the first action potential, which is 0.4 ms less than the stimulus interval; this represents a decrease in conduction latency of 0.3% (or an increase in conduction velocity of 0.3%). The 24 sympathetic efferent fibers tested with twin pulse stimulation exhibited a mean decrease in conduction latency of 0.61 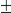 0.16%.

**Figure 3:**
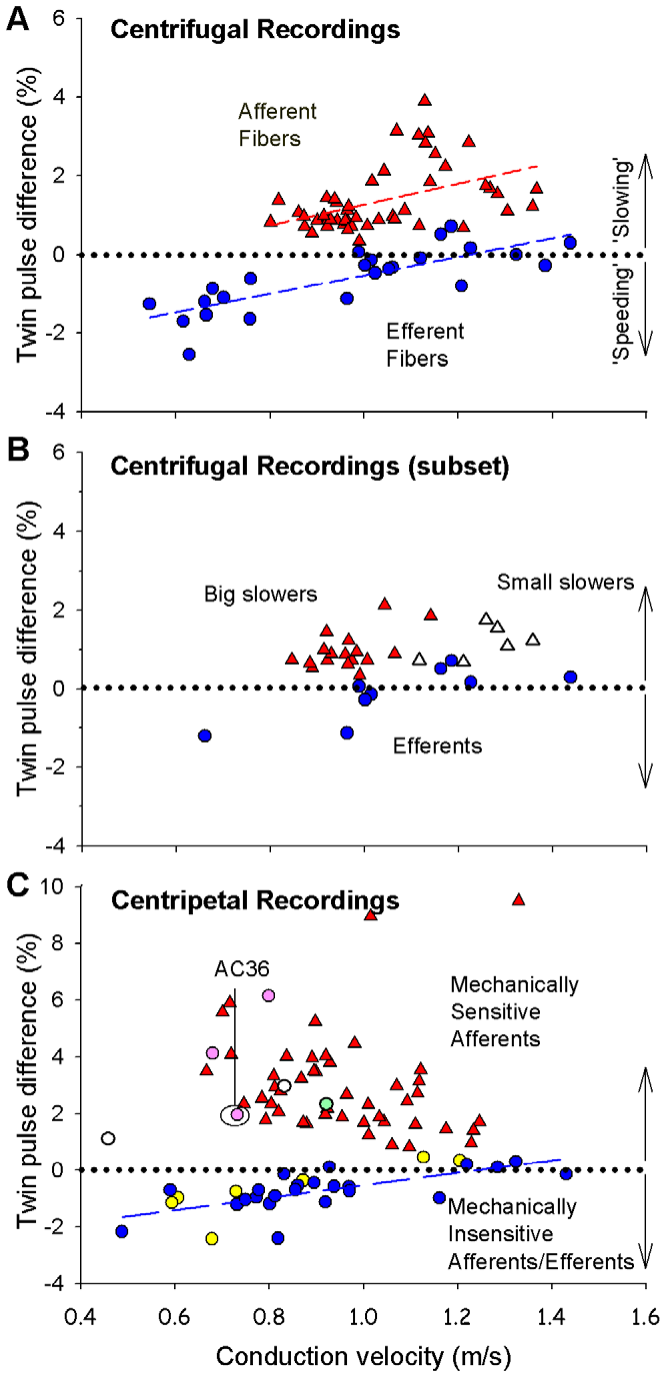
Twin pulse stimulation reveals two distinct populations. **A) Afferent and efferent fibers have different twin pulse responses.** Following electrical twin pulses (50–70 ms ISI) at the sciatic nerve, all afferent fibers (red triangles, n = 48) exhibited an increase in conduction latency for the second action potential (AP). In sympathetic efferent fibers (blue circles, n = 24) the change in conduction latency was dependent on the initial conduction velocity (R^2^  = 0.63); slower fibers showed a decrease in latency (i.e., speeding of conduction) whereas faster fibers demonstrated a modest increase in latency. The data clusters are almost non-overlapping. A negative value for twin pulse latency difference means that the second AP has a shorter latency than the first AP. **B) The initial twin pulse response does not predict the amount of activity-dependent slowing in the afferents.** The “big slowers” and “small slowers” in response to the repeated stimulation paradigm (Fig. 4) have a similar twin pulse difference but different conduction velocities. The data are from all fibers in which the activity-dependent slowing protocol (2 Hz, 3 min) was performed. **C) Mechanically-sensitive and -insensitive fibers respond differently to twin pulse stimulation.** In a separate series of experiments, the response to electrical stimulation at peripheral terminals was examined. Mechanically sensitive nociceptors (triangles) exhibited twin pulse slowing. Most mechanically-insensitive fibers behaved like sympathetic fibers (blue circles) and exhibited twin pulse speeding. In ten mechanically-insensitive fibers capsaicin and/or histamine were injected into the electrical receptive field; three responded (pink circles) and were likely afferents; seven were unresponsive (yellow circles) and are presumed sympathetic fibers. One mechanically-insensitive fiber (green circle) not tested with chemicals behaved like an afferent to the repeated twin pulse paradigm (see Fig. 6). Two mechanically-insensitive fibers (open circles) not tested with chemicals or repeated twin-pulses exhibited twin-pulse slowing and may therefore be afferents.

The change in conduction latency between the first and second action potential we call the “**twin pulse difference**.” A negative twin pulse difference corresponds to a decrease in conduction latency for the second action potential or an increase in conduction velocity (i.e., speeding). The normalized twin pulse latency difference that is plotted in the figures was computed as the difference between the latencies of the first and second AP divided by the latency of the first AP. As shown in Fig. 3A (circles), the twin pulse difference was correlated with conduction velocity (R^2^  = 0.63, p<0.001); the greatest supra-normal conduction was observed at the slowest conduction velocities.

### Afferent Fibers Exhibit Sub-Normal Conduction

In response to twin pulse stimulation at the sciatic nerve, all afferent fibers exhibited a slowing of conduction (i.e., a positive twin pulse difference); the second action potential was conducted at a slower conduction velocity than the first action potential. For the examples in Fig. 2C, the stimulus interval was 50 ms. In this recording, action potential waveform numbers 1, 2, and 3 (at latencies of 234.1, 247.6, and 251.7 ms, respectively) were positively identified as belonging to dorsal root afferent fibers. For the fiber represented by waveform # 2, the second action potential arrived at the recording electrode 52.2 ms after its first action potential, which is 2.2 ms greater than the stimulus interval; this represents an increase in conduction latency of 0.9% (or a decrease in conduction velocity of 0.9%). The other two afferent fibers in this recording (waveform # 1 and 3) also exhibited a longer latency for the second action potential (by 0.9 and 1.0%, respectively). The 48 afferent fibers tested with twin pulse stimulation exhibited a mean increase in conduction latency of 1.39 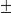 0.11% which was significantly different from that observed for sympathetic efferent fibers (p<0.001). As shown in Fig. 3A (triangles), the twin pulse difference was weakly correlated with conduction velocity (R^2^  = 0.19, p<0.01). Notably, the data for the afferent and efferent fibers appeared to form two clusters that were essentially non-overlapping (Fig. 3A).

### Activity-Dependent Slowing in Sympathetic Efferent Fibers

**Figure 4:**
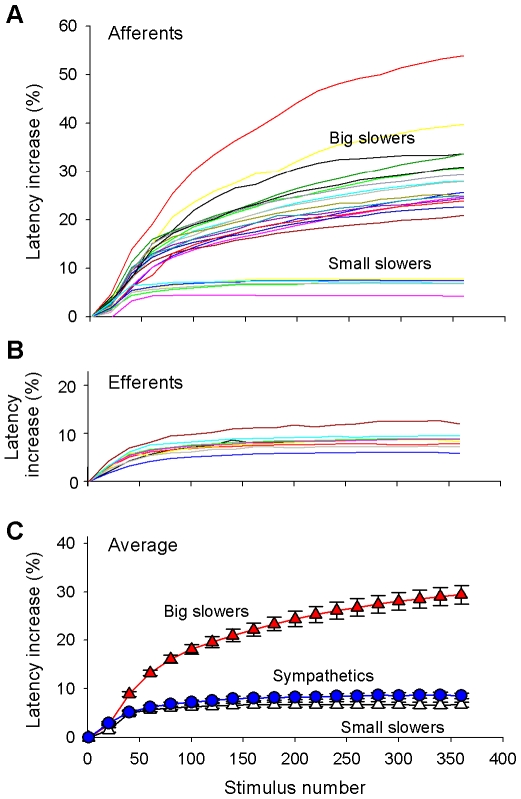
Activity dependent slowing of afferent and sympathetic-efferent fibers. **A) Afferents.** In response to the train of 360 electrical pulses (2 Hz for 3 min), the afferent fibers exhibited two types of slowing in centrifugal recordings. The “big slowers” exhibited an increasing conduction latency throughout the stimulus train and reached a final conduction latency increase of greater than 20% (n = 17). The latency of the “small slowers” increased during the first 50 pulses but reached a plateau with a final conduction latency increase less than 10% (n = 6). **B) Efferents.** In centrifugal recordings, the sympathetic fibers (n = 9) looked like the small slowers and also had a final conduction latency increase less than 10%. **C) Average activity dependent slowing for two types of afferents and for sympathetic efferents.** The “small slowing” afferents and the sympathetic fibers exhibited similar activity dependent slowing. The “big slowing” afferent fibers were significantly different. Data were obtained from centrifugal recordings.

A train of 360 stimuli (2 Hz for 3 min) was used to investigate activity-dependent slowing of conduction [17]. For the example sympathetic efferent fiber in Fig. 2A, the conduction latency increased during the first 100 pulses, but approached a plateau level for subsequent pulses (Fig. 2B); the final increase in latency was 12.9 ms which corresponded to an 8.7% increase in conduction latency. The 9 efferent fibers studied with this protocol exhibited a similar time course for their increase in conduction latency (Fig. 4B); the mean increase in latency at the end of the protocol was 8.6 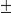 0.6% (Fig. 4C). We did not observe a reversal of the conduction latency increase near the end of the 3 min stimulation as has been reported in human efferent fibers recorded in distal nerves [20].

### Activity-Dependent Slowing in Afferent Fibers

The response of a typical afferent fiber to the 2 Hz, 3 min stimulus train is illustrated in Fig. 2D. For this fiber (corresponding to waveform # 2 in Fig. 2C), the conduction latency increased continuously throughout the stimulation; the final increase in latency was 60.7 ms which corresponded to a 24.6% increase in conduction latency. The other two afferent fibers in this recording (waveform # 1 and 3 in Fig. 2C) also exhibited pronounced activity-dependent slowing (by 22.7 and 25.0%, respectively).

The 23 afferent fibers studied with this protocol in centrifugal recordings exhibited two different types of slowing. For 17 afferent fibers (labeled as “big slowers” in Fig. 4A), the conduction latency increased continuously throughout the stimulus train (i.e., similar to that shown in Fig. 2); the mean increase in latency at the end of the protocol (29.4 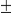 1.9%) was substantially greater than for the sympathetic efferent fibers (p<0.001). For 6 afferent fibers (labeled as “small slowers” in Fig. 4A), the time course for the increase in conduction latency was similar to that of the sympathetic efferent fibers (Fig. 4B). The mean increase in latency at the end of the protocol for these afferents (6.7 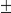 0.5%) was significantly (but not substantially) smaller than for the sympathetic efferents (p<0.05).

The “big slowers” and “small slowers” did not differ with regard to their latency difference following twin pulse stimulation (Fig. 3B). However, as can be seen from Fig. 3B, the mean conduction velocity of the small slowers was significantly faster (1.25 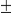 0.3 m/s) than the big slowers (0.96 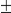 0.5 m/s, p<0.05).

### Centripetal Recordings Following Electrical Stimulation at the Cutaneous Terminals

In a separate series of experiments, twin-pulse electrical stimulation (ISI  = 40–70 ms) was applied to the cutaneous terminals of 46 mechanically-sensitive nociceptive afferent fibers (MSAs) and of 36 mechanically-insensitive afferent/efferent fibers. As shown in Fig. 3C (triangles), all of the MSAs exhibited a slowing of conduction to stimulation with a single twin-pulse. The average twin pulse difference (3.0 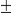 0.3%) was greater than observed for the afferent fibers in the centrifugal recordings with proximal sciatic nerve stimulation (Fig. 3A, p<0.001). This suggests that the twin-pulse slowing for afferent fibers is more pronounced in the cutaneous arbor than in the proximal parent axon.

In contrast, most of the mechanically-insensitive fibers exhibited a speeding to the twin pulse stimulation (circles, Fig. 3C). This speeding was similar to that observed for the sympathetic efferent fibers (Fig. 3A) in the centrifugal recordings.

**Figure 5:**
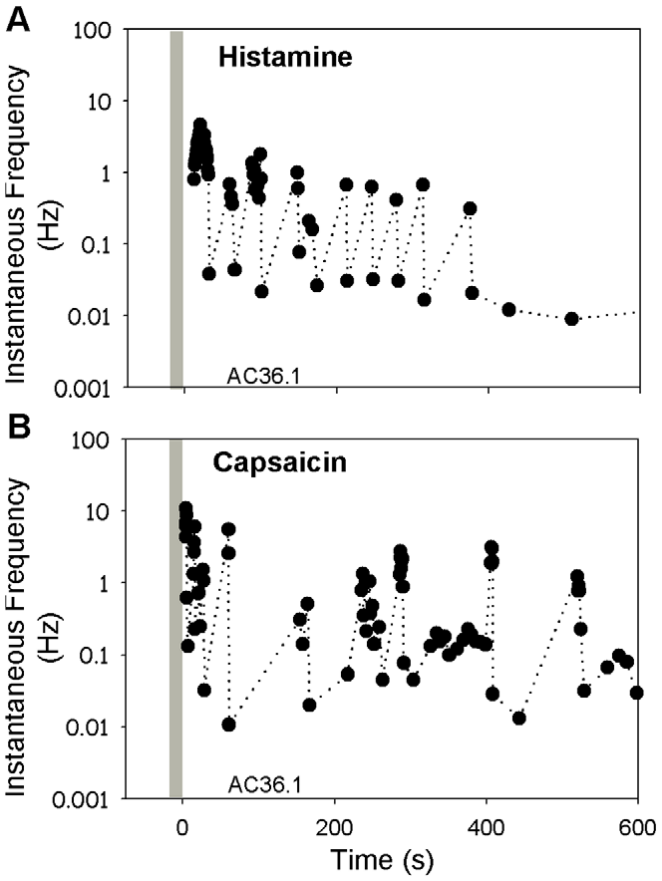
Mechanically-insensitive afferent fiber responds to histamine and capsaicin. The C-fiber mechanically-insensitive afferent identified as AC36 in Fig. 3Cresponded to intradermal injection of histamine (10 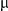
g/10 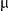
l) and capsaicin (10 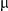
g/10 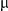
l). The response to both chemicals was greatest during the first 30 s and lasted about 10 min. Instantaneous frequency is plotted as a function of time following injection (each dot corresponds to time of an action potential). Note, there was no response during needle insertion or drug injection (shaded bar).

To test whether these mechanically-insensitive fibers were afferents, we injected histamine (10 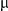
g/10 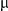
l) and/or capsaicin (10 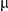
g/10 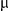
l) into the electrical receptive field in 10 of these fibers. Seven did not respond to histamine or capsaicin, but three of these mechanically-insensitive fibers did respond and are likely mechanically-insensitive afferents. The responses of one of these fibers (labeled AC36 in Fig. 3C) to the intradermal injection of histamine and capsaicin are shown in Fig. 5. Notably, the three mechanoinsensitive fibers responsive to histamine/capsaicin (pink circles in Fig. 3C) exhibited twin-pulse slowing comparable to that seen in the mechanically-sensitive afferents (red triangles in Fig. 3C), whereas the seven chemically unresponsive fibers (yellow circles in Fig. 3C) exhibited twin-pulse speeding comparable to the presumed sympathetic fibers (blue circles in Fig. 3C). While conduction velocities of different groups of fibers overlapped, differences in latencies after twin pulse stimulation in mechanoinsensitive afferents and presumed sympathetic fibers did not, i.e. the change in latency following twin pulse stimulation differentiates mechanoinsensitive afferents from sympathetic efferent fibers.

### Supra-Normal Conduction Is Dependent on Stimulus Interval

In centripetal recordings we used the 60 twin pulses protocol to investigate the relationship of activity-dependent slowing and relative speeding of conduction. A subset of fibers was tested with twin pulse stimulation where pulses of twin stimuli were separated by a fixed stimulus interval (10, 20, 50, 100, 200 or 500 ms). In response to the first twin pulse, two outcomes were observed: 1) a negative twin pulse difference that indicates supra-normal conduction of the second action potential relative to the first action potential, or 2) a positive twin pulse difference indicating a slowing of conduction. Based on our centrifugal recordings, a fiber was presumed to be a sympathetic efferent if it showed a negative twin-pulse difference to the first twin pulse at 50-70 ms stimulus interval and was unresponsive to noxious mechanical or chemical stimuli. Units responsive to mechanical stimuli were categorized as MSA fibers.

**Figure 6:**
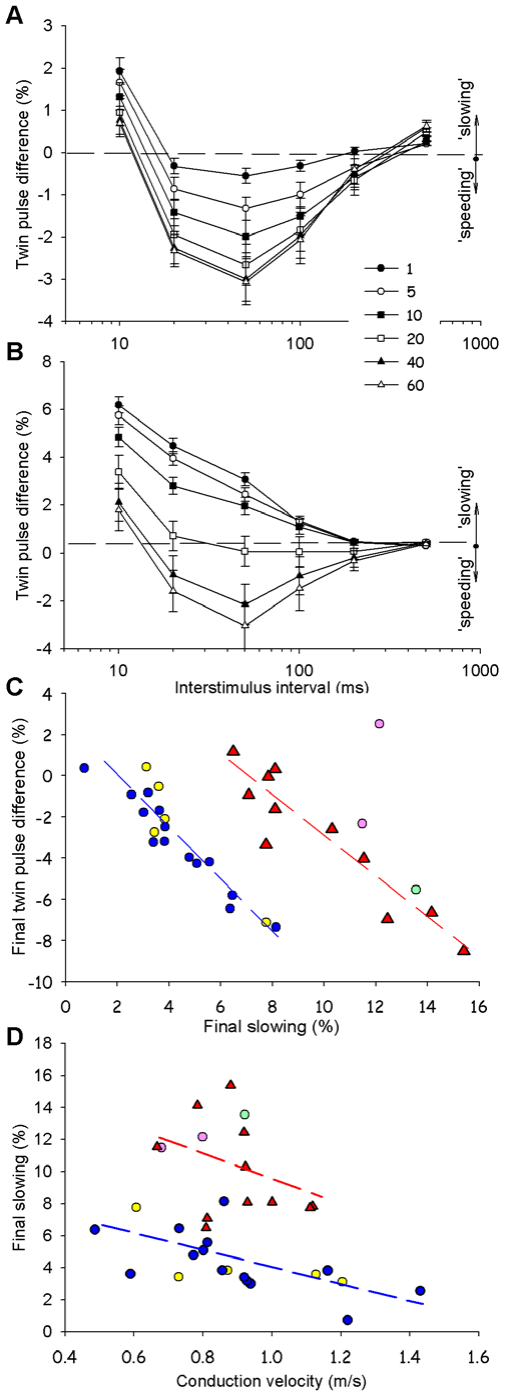
Mechanically-sensitive afferents and presumed sympathetic efferents exhibit different twin pulse speeding and activity-dependent slowing during the repeated twin-pulse paradigm. **A) Presumed sympathetic efferents.** The twin-pulse latency difference is plotted versus stimulus interval for the 1^st^, 5^th^, 10^th^, 20^th^, 40^th^ and 60^th^ twin stimulus. Twin pulse speeding is observed for ISIs between 20 and 100 ms. The amount of twin pulse speeding increases during the repeated paradigm (n = 13–20, except at the 1^st^ twin pulse at 50 ms where n = 32). **B) Mechanically-sensitive afferents.** For the first twin pulse, twin pulse slowing is observed over all ISIs. After 20 twin pulses, speeding develops over a range of ISIs from 20 to 100 ms. (n = 7–12, except at the 1^st^ twin pulse at 50 ms where n = 46). **C) The plot of final twin pulse difference versus final slowing reveals two non-overlapping clusters corresponding to the presumed sympathetic efferents and the afferents.** Presumed sympathetic efferents and afferents exhibited a similar range of twin-pulse speeding by the final (60^th^) twin pulse of the repeated twin pulse protocol. The final activity-dependent slowing (i.e., increase in latency of the first AP in the last twin-pulse relative to the latency of the first AP in the train) was greater in the sympathetics than the afferents. One mechanically-insensitive fiber (green circle) not tested with chemicals behaved like an afferent. Only data from twin pulses separated by 50 ms are included. Symbol coding same as in Fig. 3C. **D) Correlation of the final slowing and conduction velocity.** The final slowing tends to decrease as the conduction velocity increases for both classes of fibers. Mechanically-sensitive afferents and presumed sympathetic efferents form distinct clusters. Note that the green circle is again within the afferent cluster and presumably a mechanically-insensitive afferent. All data in this figure were obtained in centripetal recordings.

For the presumed sympathetic fibers, a speeding of conduction was observed to the first twin pulse over stimulus interval range of 20 to 100 ms, with slowing of conduction at ISIs of 10 ms and 500 ms (filled circles in Fig. 6A). The greatest speeding occurred at the 50 ms ISI.

For the MSA fibers, slowing of conduction (i.e., positive twin pulse difference) was observed to the first twin pulse over the entire range of stimulus intervals (filled circles in Fig. 6B). The largest twin pulse slowing (6.2 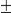 0.3%) occurred for the10 ms ISI, and the amount of slowing decreased as the ISI increased.

### Activity-Dependent Slowing Potentiates Twin-Pulse Speeding

Previous investigators have suggested that a relative speeding of conduction to twin pulse stimulation develops in C-fiber nociceptors in the presence of activity-dependent slowing [9]. To investigate this phenomenon, we delivered 60 twin pulses (Fig. 1C) to the cutaneous terminals of MSAs and presumed sympathetic fibers. Typical results from this protocol are illustrated in Text S1 (see also Fig. S2 and S3).

In both classes of fibers, the repeated twin pulses led to the development of activity-dependent slowing of the first action potential of the twin pulse. For the presumed sympathetic fibers, the activity-dependent slowing increased quickly (time constant  = 27 s, Fig. S4B) and reached a plateau within the first 20 pulses. In contrast, the mechanically-sensitive afferents exhibited a continuous increase in activity -dependent slowing throughout the protocol (time constant  = 64 s, Fig. S4B).

Fig. 6A illustrates the average twin pulse data from the presumed sympathetic fibers. Several findings are noteworthy: 1) At the smallest ISI (i.e.,10 ms), only twin-pulse slowing was observed. With increasing number of twin stimuli, the twin pulse difference became smaller but remained positive throughout the stimulus train. 2) At the largest ISI (i.e., 500 ms), only twin-pulse slowing was observed. The magnitude of the twin pulse difference did not vary with increasing twin pulse stimuli. 3) Only small relative speeding was observed when twin pulses separated by 200 ms were applied. 4) For ISIs between 20 and 100 ms, speeding was present for the first twin pulse, and the amount of speeding increased as the number of twin pulses increased, but reached a plateau after about 20 twin pulses (see also Fig. S4C). 5) The largest twin pulse speeding occurred at the 50 ms ISI. This was true throughout the repeated stimulation protocol. The maximum speeding (3.1 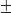 0.5%) was observed at the end of stimulation at an ISI of 50 ms.

The average data for all C-MSA fibers are shown in Fig. 6B. Several findings are noteworthy: 1) Regardless of the stimulus interval used, no speeding was observed for the first twin pulse. 2) At the smallest stimulus interval (i.e.,10 ms), only slowing was observed. With increasing number of twin stimuli, the magnitude of the twin pulse difference became smaller but remained positive throughout the stimulus train. 3) For intervals between 20 and 100 ms, slowing was present for the first 20 twin pulses. After 20 twin pulses, twin pulse speeding developed and the magnitude of the speeding continued to increase for the remainder of the twin pulses (See also Fig. S3C). 4) The largest speeding occurred at the 50 ms stimulus interval; the maximum value for the speeding in MSAs (3.0 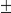 1.0%) occurred at the end of the train and did not differ from the speeding in presumed sympathetic fibers (p>0.05).

### Final Twin-Pulse Speeding Is Correlated with Final Activity-Dependent Slowing

Previous microneurography studies in humans [21] suggested that relative speeding in C- fiber nociceptors is correlated with the amount of slowing of the first action potential. To investigate if such a relationship is also seen in C- fibers of non-human primate, the twin pulse difference of final twin stimulus (twin pulse #60) was plotted against the total amount of slowing for the first action potential of the final twin pulse (i.e., the latency difference between action potential #119 and action potential #1). Only data from stimulation with an ISI of 50 ms were used. Fig. 6C summarizes these data for the different classes of fibers that were investigated in this study. In both classes of C- fibers, the amount of twin-pulse speeding at the end of the stimulus train was correlated with the amount of total slowing at the end of the stimulation. The average final slowing for the mechanically-sensitive afferents (9.9 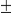 0.9%) was significantly greater than for the presumed sympathetic fibers (4.3 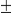 0.4%, p<0.001), but, as noted above, the average amount of final twin pulse speeding did not differ. Notably, the presumed efferent fibers and afferent fibers formed two non-overlapping clusters.

The latency difference after the final twin pulse latency was correlated to the difference after the first twin pulse (Fig. S5). Thus, sympathetic fibers that exhibited more initial twin pulse speeding developed more twin pulse speeding during the repeated stimulation than fibers with little initial twin pulse speeding.

A plot of final slowing versus conduction velocity (Fig. 6D) reveals two partially overlapping clusters. The fiber marked by the green circle is in the middle of the afferent cluster in figures 6C, 6D, and 3C, and thus likely is a mechanically-insensitive afferent.

## Discussion

In the peripheral nerve, unmyelinated sympathetic efferent fibers and unmyelinated primary afferent fibers are known to exhibit different compositions of voltage gated K+ and Na+ ion channels. This suggests that their conduction properties may differ. Using centripetal and centrifugal recordings from peripheral nerves, we found that activity-dependent slowing of conduction to repeated electrical stimulation was typically less in sympathetic fibers than in nociceptive afferent fibers. An important novel finding was that sympathetic efferent fibers in cutaneous nerves exhibit a speeding of conduction to a single pair of electrical stimuli separated by 50 ms; the second action potential was conducted faster than the first unconditioned action potential. This was never observed in unmyelinated afferent fibers. This difference provides a criterion by which sympathetic efferents and unmyelinated afferents can be differentiated in peripheral nerve recordings.

### Unmyelinated Nerve Fibers Exhibit Different Patterns of Conduction Velocity Slowing upon Repetitive Electrical Stimulation

In centrifugal recordings from sciatic nerve fibers that were positively identified as afferent or efferent using collision techniques, we found different conduction slowing patterns in response to 2 Hz repetitive stimulation (3 min). Afferent fibers exhibited two distinct patterns: one afferent group (‘large’ slowers) slowed by ≥20% of the initial conduction velocity, whereas a second afferent group (‘small’ slowers) slowed significantly less (∼7%). Such a dichotomy is in agreement with previous studies in rat [7], [22], [6] and human [17], [10], [23] in which the first group likely consists of nociceptive afferents, and the second group of ‘cold’ fiber afferents. The fibers in this study that were positively identified as sympathetic efferents exhibited a slowing that was similar to the second group of afferents; this finding is consistent with observations made in microneurographic recordings in human [20].

There are, however, differences between previous studies and the present study: 1) In recordings from human peripheral nerve, about 35% of fibers showed pronounced slowing (≥30%) [17], but in the present study less than 25% of unmyelinated afferent fibers showed such slowing in centrifugal recordings. However, we applied electrical stimuli to the proximal nerve trunk and not to cutaneous peripheral terminals which have been suggested to contribute mainly to conduction slowing. Thus a difference in stimulation site may account for these differences. 2) We did not observe unmyelinated afferents with a ‘type 3′ slowing that have been hypothesized to represent low threshold C fiber afferents and that are characterized by only a small increase (<1%) in latency during ongoing stimulation (2 Hz, 3 min). However, the incidence of such afferents is small (∼5–6%) [17], [11], and they may have been missed in our centrifugal recordings.

Previous investigators postulated that activity dependent hyperpolarization associated with the Na+/K+ pump accounts for conduction slowing in unmyelinated afferents during repetitive electrical stimulation [14], [9], [11]. However, blockade of Na+/K+ ATPase has recently been demonstrated to increase conduction slowing [12]. In addition, the degree of use-dependent slowing of conduction was not affected by blocking TTX sensitive currents with low doses of tetrodotoxin (TTX). Instead, the use dependent slowing was sensitive particularly to lidocaine and carbamazepine, the two sodium channel blockers that have been known for their greater effectiveness in blocking slow inactivated NaV1.8- than TTX sensitive channels. Based on these data the authors suggest that conduction slowing upon repetitive electrical stimulation is due to slow inactivation of TTX resistant sodium channels, especially NaV1.8, and that hyperpolarization induced by activity in Na+/K+ ATPase rescues sodium channels from inactivation [12]. Differences in activity dependent slowing are likely due to differences in sodium channel expression in axonal membranes of the different C fiber populations. For example, dorsal root ganglion neurons, but not superior cervical ganglion neurons, express the TTX resistant sodium channel NaV1.8 [24] which has been suggested to underlie activity dependent slowing in nociceptive afferents because this channel more readily enters a slow inactivated state upon activation and recovers slowly from that inactivation [13], [25].

### Absolute Speeding Distinguishes Sympathetic Efferents from Unmyelinated Afferents

In centrifugal recordings, we observed that sympathetic efferents, but not primary afferents, exhibited absolute speeding of conduction when tested with a single twin pulse (ISI 50–70 ms); the conduction velocity of the second action potential was faster than the first, naive action potential. Similarly, in centripetal recordings, absolute speeding of conduction to a single twin pulse was observed in nerve fibers that were unresponsive to mechanical, thermal or chemical stimuli. Because of these features it is likely that these unmyelinated fibers are sympathetic efferent fibers. In contrast, mechanosensitive fibers only showed slowing of conduction to a single twin pulse. In addition, mechanoinsensitive fibers that were responsive to chemical stimuli and hence were likely afferents, exhibited conduction slowing in response to a single twin pulse. Only a few chemosensitive, mechanoinsensitive afferents were studied in centripetal recordings, and we therefore cannot exclude the possibility that some groups of mechanoinsensitive afferents (e.g. cold nociceptive afferents), which we did not encounter in centripetal recordings, will show absolute speeding to twin pulse stimulation. This possibility, however, seems unlikely, since none of the fibers identified as afferents in the centrifugal recordings showed absolute speeding to twin pulses stimulation. Absolute speeding following a single twin pulse appears to be a distinct feature of mechanoinsensitive, efferent nerve fibers. Similar observations have recently been made in recordings from peripheral nerve fibers in pig (O. Obreja and M Schmelz, personal communication). Furthermore, absolute speeding to a single twin pulse (ISI 50 ms) is also likely to be present in sympathetic nerve fibers of human and rat since recovery cycles of conduction velocity in sympathetic nerve fibers in these species show an increase in conduction velocity at interspike intervals below 150 ms regardless of mean stimulation frequency [26], [11].

Following an action potential, the axonal membrane of unmyelinated nerve fibers undergoes time dependent excitability changes as originally proposed by Barrett and Barrett [27] and supported by recent findings in human [14], [9] and rat [11]; this after-potential in unmyelinated axons appears to reflect a residual charge on the membrane which decays exponentially through the membrane resistance-capacitance (RC) circuit. The size and polarity of this charge is dependent on the voltage-dependent gating mechanism of ion channels. For example, a depolarizing after-potential occurs if the outward potassium movement does not quite compensate for the inward sodium movement leading to excess intracellular sodium; this would lead to a “hyperexcitability” of the membrane and a speeding of conduction as seen in the sympathetic fibers. A hyperpolarizing after-potential occurs when the outward potassium current exceeds the inward sodium movement; this would lead to a “hypoexcitability” of the membrane and a slowing of conduction as seen in the afferent fibers. Our findings are consistent with a previous study [28] reporting a ‘positive’ (i.e. hyperpolarizing) afterpotential in unmyelinated vagal afferents and a ‘negative’ (i.e. depolarizing) afterpotential in sympathetic fibers of the hypogastric nerve. The difference between afferents and efferents (for a single twin pulse) may reflect a difference in the potassium/sodium channels that are expressed in afferents and sympathetic efferents. For example, NaV1.8 is not expressed in sympathetic fibers but is expressed in nociceptive afferents. In addition, DRG neurons express higher levels of Kv1.1, Kv 1.8, and Kv 3.1 than sympathetic neurons (Munns and Koltzenburg, personal communication). The depolarizing afterpotential in sympathetic neurons may also be due to activation of Cl- channels via voltage-dependent Ca++ channels [29].

### Relative Speeding Develops in Afferent Fibers

During the course of the repeated twin-pulse stimulation, the twin pulse speeding became more pronounced in the efferent fibers. The afferent fibers, which initially showed twin-pulse slowing, developed a relative twin-pulse speeding (e.g., Fig. S2). Sympathetic efferents exhibited absolute speeding of the second action potential when pulses of twin stimuli were separated by intervals of 20–100 ms. This speeding increased with ongoing stimulation but reached a plateau after about 20 twin pulses. In contrast, mechanosensitive afferents never showed absolute speeding regardless of the stimulus interval used; however, relative speeding developed during ongoing stimulation at stimulus intervals of 20–100 ms. Thus, at these stimulus intervals, the axonal hypoexcitability which is apparent as conduction slowing of the trailing action potential at the beginning of the stimulation decreases and is replaced by axonal hyperexcitability that becomes evident as relative speeding. At the end of the stimulation with 60 twin pulses, slowing of the leading action potential was significantly larger in afferent than in sympathetic efferent fibers. In both fiber classes, the latency difference between the leading and trailing action potential correlated negatively with the increase in latency of the last leading action potential. Furthermore, the time course for the slowing of the leading action potential was similar to the time course for the speeding of the trailing action potential for both the sympathetics and the afferents (Fig. S4B,C). Taken together these findings suggest that related mechanism(s) may underlie the phenomena of activity-dependent slowing and relative ‘speeding’.

As hypothesized earlier [9], [11], activity dependent hyperpolarization increases sodium influx and reduces potassium outflux during an action potential thereby creating a net depolarizing charge on the axonal membrane and resulting in a depolarizing afterpotential. Our observation that slowing and relative speeding have a similar time course is in agreement with this hypothesis.

The exponential recovery from relative speeding for both the afferents and the sympathetic efferents is consistent with a discharge through the membrane RC circuit. The time constants for recovery for the sympathetics (137 ms) and the afferents (89 ms) are comparable to those reported previously [9], [11].

### Conduction Criterion to Distinguish Afferents from Sympathetic Efferents

A fiber that is insensitive to mechanical stimuli applied to its cutaneous terminals can be either a sympathetic efferent or a mechanically-insensitive afferent. Positive classification of an afferent requires the identification of the adequate stimulus which may be one of many noxious chemicals (e.g., mustard oil, capsaicin, histamine) or thermal stimuli (cold, heat). To avoid applying multiple noxious agents to the skin, it would be useful to have an independent criterion for identifying sympathetics. Our finding that only sympathetic efferents show absolute speeding of conduction to a twin pulse (ISI  = 50 ms) provides such a criterion.

## Supporting Information

Figure S1 Voltage-latency curve for an afferent obtained by electrical stimulation at the receptive field using the well electrode. Before starting with the electrical stimulation protocols at the cutaneous terminals, electrical stimuli of constant duration (1 ms) but of increasing intensity were applied every 4 s. Threshold for electrical activation and the latency at this stimulus intensity were measured. Intensity was increased until a step decrease in conduction latency was observed. The stimulus intensity necessary to produce this step and the resulting new latency were noted. Intensity was increased to the upper limit of the Grass constant-voltage stimulator (150 V) or the Digitimer Constant Current stimulator (100 mA). Stimulus intensities and conduction latencies were used to generate voltage-latency curves similar to the example shown in this figure. At this stimulation site, the electrical threshold for activation was 60 V, and the resulting conduction latency was about 125 ms. Up to an intensity of 85 V, the conduction latency was stable, but at an intensity above 85 V, the latency stepped down to about 123 ms. Another latency step was observed with stimulus intensities above 99 V at which the latency decreased to about 115 ms. No additional decrease in conduction latency was observed up to an intensity of 150 V. These different latency levels correspond to discrete action potential initiation sites within the cutaneous arbor of the afferent. The purpose of these voltage-latency curves was to identify a wide stimulus intensity window over which the latency of the unit under study was stable to insure that the AP initiation site was fixed. For the subsequent electrical test protocols, stimulus intensity was usually set half way between the upper and lower limits of such a stable window. For this particular fiber, the stimulator was set at 125 V.
(0.01 MB PDF)

Figure S2 Presumed sympathetic fiber shows absolute and relative speeding to 60 twin pulses. A recording from a C fiber is schematically summarized in Fig. 2A. For this presumed sympathetic fiber, transcutaneous electrical stimulation consisted of twin pulses delivered every 2 s with a stimulus interval between pulses of a twin stimulus of 50 ms. In response to twin stimulus #1, the first action potential (AP) arrived at the recording electrode 123 ms after the delivery of the first pulse at the cutaneous receptive field (see left vertical dashed line). Since the stimulus interval was 50 ms, the 2nd AP was expected to arrive at the recording electrode at least 173 ms after the first pulse. In other words, the 2nd AP was expected to arrive at the recording electrode about 50 ms following the 1st AP (indicated by the right vertical dashed line in Fig. 2A). However, the 2nd AP arrived at the recording electrode 171.8 ms after the first pulse, corresponding to a latency of 121.8 ms from the second pulse. Thus, the conduction of the 2nd AP was 1.2 ms faster than the conduction of the “naïve” AP (defined here as the first AP in the stimulus train). This phenomenon represents absolute “speeding” of conduction by 1% (marked by “A” in the trace for twin stimulus #1). For the second twin stimulus, the latency of the 1st AP (i.e., AP #3 in train) increased, as did the latency of the 2nd AP (AP #4). However, the latency of AP#4 was still smaller than the latency of the naive AP, indicating that absolute speeding still occurred. The relative latency of the 1st AP (S in figure) and the 2nd AP continued to increase during the course of the stimulation. At the end of the stimulus train (i.e., twin stim #60), the latency of the 1st AP (i.e., AP# 119) had increased to 129 ms (corresponding to a slowing of 4.9% relative to the naïve AP), whereas the latency of the 2nd AP (i.e., AP#120) had only increased to 124 ms. The latency of the 2nd AP was still shorter than the latency of the 1st AP, but it was now longer than the latency of the naive AP. Thus, the 2nd AP no longer showed absolute speeding, but it did show relative speeding with respect to the 1st AP (marked by “R” in the trace for twin pulse #60). The latencies of the two APs are plotted as a function of twin stimulus number in B. The latency of the naive AP (i.e., AP #1) is indicated by the horizontal line in the graph. The latency of the 2nd AP was shorter than the latency of the naive AP up to twin stimulus #14, indicating the presence of absolute speeding (see arrow labeled “A”). Throughout the stimulus train, the latency of the 2nd AP was smaller than the latency of the 1st AP, indicating the presence of relative speeding (see arrow marked “R”). The relative latency of the 1st AP (S) increases throughout the protocol indicating activity-dependent slowing. In C, the difference between the latency of the 2nd AP and the naive AP is plotted as a function of the twin stimulus number (filled symbols). For the first 14 pairs of twin pulses, this difference was negative indicating absolute speeding. For the remaining twin stimuli, this difference became positive as the latency of the 2nd AP in a given twin stimulus became larger than the latency of the naive AP. C also shows the latency difference between the 2nd AP and the 1st AP for every twin stimulus (i.e., the latency difference between twin APs, open symbols). Since the 2nd AP of a given twin stimulus always had a smaller latency than the preceding 1st AP of the same twin stimulus, the latency difference between twin APs was always negative, indicating the presence of relative speeding throughout the stimulation. As can be seen, this difference became more negative during the first 25 pairs of twin stimuli after which it reached a plateau. As shown in B, this plateau is due to the fact that the conduction latencies of both action potentials had reached a plateau by the 25th pair of twin stimuli, and that they increased only minimally throughout the rest of the train. To analyze the latency data across different fibers, the latency difference between twin APs of a given twin stimulus was normalized by dividing by the latency to the naive AP of that stimulus train and is referred to as the “twin pulse difference.” In D, the twin pulse difference data for this fiber are plotted as a function of stimulus intervals (10, 20, 50, 100, 200 and 500 ms). Each curve corresponds to data collected at a given twin stimulus number during the different trains (i.e., twin stimulus number 1, 10, 20, 40, 60). In this fiber, the stimulus intervals of 10 and 500 ms resulted only in positive latency differences, indicating that the 2nd AP was always conducted slower than the 1st AP of the twin stimulus. Moreover, the positive difference did not change markedly from the first to the 60th twin stimulus. In contrast, the latency differences between twin APs collected at the stimulus intervals of 20–200 ms were all negative. Specifically, this difference was negative with the first twin stimulus, i.e., absolute speeding was observed at these intra twin pulse intervals. Furthermore, the latency difference between twin APs became more negative for the first 20 twin stimuli, after which the difference reached a plateau and no further increase was observed. C-fiber mechanosensitive nociceptive afferent shows only relative speeding (E-H). The right hand column shows a recording in a typical C-mechanosensitive afferent (C-MSA). The interval between the twin pulses was 50 ms, and twin stimuli were applied every 2 s. As shown in E, the second AP in response to twin stimulus #1 was conducted slower than the naive AP. With ongoing stimulation, the latencies of both APs increased, i.e., the conduction for both action potentials slowed. However, at the end of the stimulus train, the second AP (AP # 120) occurred less than 50 ms after the first AP (AP #119), indicating relative speeding of the second AP. When the latencies for the first and second action potential are plotted separately (see Fig. 2F), the development of relative speeding becomes more evident. For the first 11 twin stimuli, the latency of the second AP was greater than the latency of the naive AP which is marked by the horizontal line. In addition, the latency of the second AP was larger than the latency of its preceding AP. At twin stimulus #12 the latencies from both APs were almost identical. After the 12th twin stimulus, the latency of the first AP became larger than the corresponding second AP, i.e., relative speeding (R) developed. At the end of the stimulus train, the latency of the first AP had increased by a total of 20 ms to 186 ms, whereas the latency of the second AP had only increased by 9 ms. Fig. 2G shows the latency differences between the second AP of a twin stimulus and the naive AP (filled symbols). Since the second AP was always conducted slower than the naive AP, this difference was always positive. This is in contrast to the latency difference between the second and its preceding AP of a given twin stimulus which is represented by the open symbols in Fig. 2G. In the beginning of the stimulus train, this difference was positive, but with ongoing stimulation, it became smaller. With twin stimulus #12, this difference became negative, again illustrating the development of relative speeding. Relative speeding increased over the stimulus train and at the end of the stimulation the second AP was conducted 14 ms faster than the preceding AP. Fig. 2H summarizes the normalized data that were collected for the different intra twin pulse intervals used. As can be seen, the latency difference between the twin APs of the twin stimulus #1 was always positive regardless of the stimulus interval, i.e., slowing of the second AP was always observed. Between twin stimuli 10 and 20 the difference became negative indicating the development of relative speeding. For all stimulus intervals between 20–200 ms, relative speeding increased with the number of twin stimuli applied. No major changes in conduction latency were observed when the stimulus interval was set at 500 ms.
(0.09 MB PDF)

Figure S3 Twin pulse data from mechanically-sensitive afferents and presumed sympathetic efferents. Fifteen presumed sympathetic fibers and 10 mechanically-sensitive afferents were studied with 2 or more different stimulus intervals using the repeated twin pulse paradigm. The twin pulse difference for the first twin pulse (A and B) and the last twin pulse (C and D) are plotted for these presumed sympathetic fibers (A and C) and mechanically-sensitive afferents (B and D). Each line corresponds to a different fiber.
(0.07 MB PDF)

Figure S4 Time constants. A. Recovery from twin pulse speeding. The average twin pulse difference for the last twin pulse is plotted as a function of stimulus interval for the mechanically-sensitive afferents (red triangles) and the presumed sympathetic fibers (blue circles). Exponential fits to the data over the interval from 50 to 500 ms are indicated by the dashed curves. The recovery time constant for the mechanically-sensitive afferents (89 ms) was shorter than for the presumed sympathetic fibers (137 ms). B. Activity-dependent slowing during the repeated twin pulse stimulation. The relative increase in latency of the first action potential in the twin pulse is plotted as a function of time during the twin pulse paradigm that lasted for 120 s for the mechanically-sensitive afferents (red triangles) and the presumed sympathetic fibers (circles). Exponential fits to the data are indicated by the dashed curves. Most of the activity-dependent slowing of the sympathetic fibers occurred in the first 20 pulses (time constant  = 27 s). The activity-dependent slowing in the mechanically-sensitive afferents continued to increase throughout the paradigm (time constant  = 64 s). The activity-dependent slowing of the first action potential was not dependent on the ISI of the twin pulse. C. Twin-pulse speeding during the repeated twin-pulse paradigm. The twin-pulse difference is plotted as a function of time during the twin-pulse paradigm. Average data for the 50 ms stimulus interval are used. For the presumed sympathetic fibers (blue circles), the twin-pulse difference starts negative (i.e., speeding); the magnitude of the twin-pulse difference increases for the first 20 pulses and then reaches a plateau (time constant  = 21 s). For the mechanically-sensitive afferents (red triangles), the twin-pulse difference starts positive (i.e., twin-pulse slowing); the twin-pulse difference decreases throughout the paradigm reaching a negative value (i.e., speeding) comparable to the sympathetic fibers by the end of the paradigm (time constant  = 83 s).
(0.06 MB PDF)

Figure S5 First twin pulse latency correlates with last twin pulse latency. This scatter plot of first twin pulse latency versus last twin pulse latency (i.e, to the 60th twin pulse in the train) reveals a correlation for the sympathetic fibers (circles) (R2  = 0.59, p<0.001) and the afferent fibers (triangles) (R2  = 0.34, p = 0.058). Thus, sympathetic fibers that exhibited more initial twin pulse speeding developed more twin pulse speeding during the repeated stimulation than fibers with little initial twin pulse speeding.
(0.03 MB PDF)

Text S1 Supplemental material.
(0.05 MB DOC)
