# Supplementary figures and images for: Conduction Properties Distinguish Unmyelinated Sympathetic Efferent Fibers and Unmyelinated Primary Afferent Fibers in the Monkey
Source: PLoS One. 2010 Feb 5;5(2):e9076. doi: 10.1371/journal.pone.0009076 (PMC2816714; doi:10.1371/journal.pone.0009076)

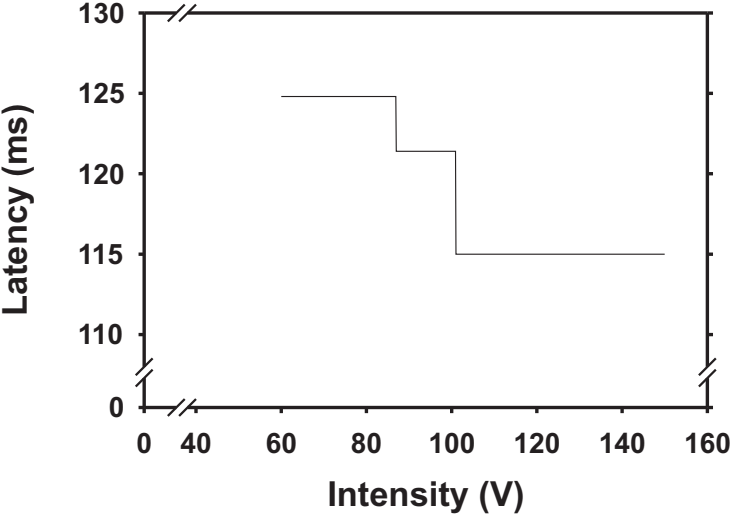

Fig. S1

Supplement: Figure S1 — Voltage-latency curve for an afferent obtained by electrical stimulation at the receptive field using the well electrode. Before starting with the electrical stimulation protocols at the cutaneous terminals, electrical stimuli of constant duration (1 ms) but of increasing intensity were applied every 4 s. Threshold for electrical activation and the latency at this stimulus intensity were measured. Intensity was increased until a step decrease in conduction latency was observed. The stimulus intensity necessary to produce this step and the resulting new latency were noted. Intensity was increased to the upper limit of the Grass constant-voltage stimulator (150 V) or the Digitimer Constant Current stimulator (100 mA). Stimulus intensities and conduction latencies were used to generate voltage-latency curves similar to the example shown in this figure. At this stimulation site, the electrical threshold for activation was 60 V, and the resulting conduction latency was about 125 ms. Up to an intensity of 85 V, the conduction latency was stable, but at an intensity above 85 V, the latency stepped down to about 123 ms. Another latency step was observed with stimulus intensities above 99 V at which the latency decreased to about 115 ms. No additional decrease in conduction latency was observed up to an intensity of 150 V. These different latency levels correspond to discrete action potential initiation sites within the cutaneous arbor of the afferent. The purpose of these voltage-latency curves was to identify a wide stimulus intensity window over which the latency of the unit under study was stable to insure that the AP initiation site was fixed. For the subsequent electrical test protocols, stimulus intensity was usually set half way between the upper and lower limits of such a stable window. For this particular fiber, the stimulator was set at 125 V. (0.01 MB PDF) [file pone.0009076.s001.pdf]

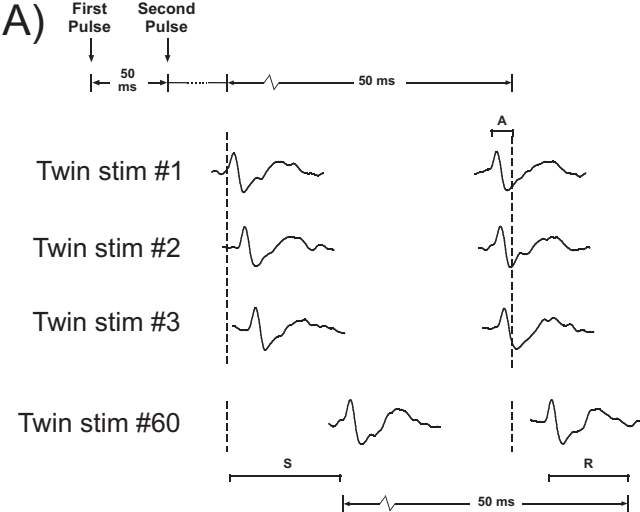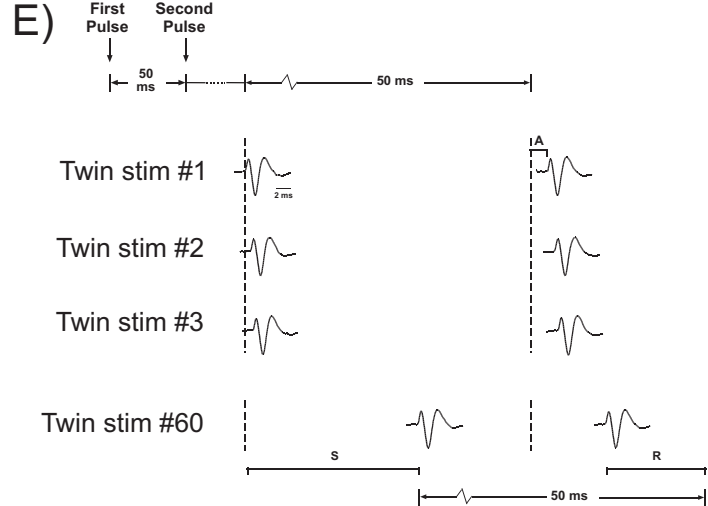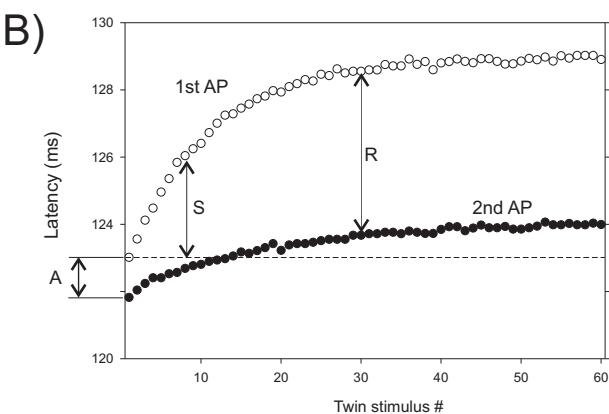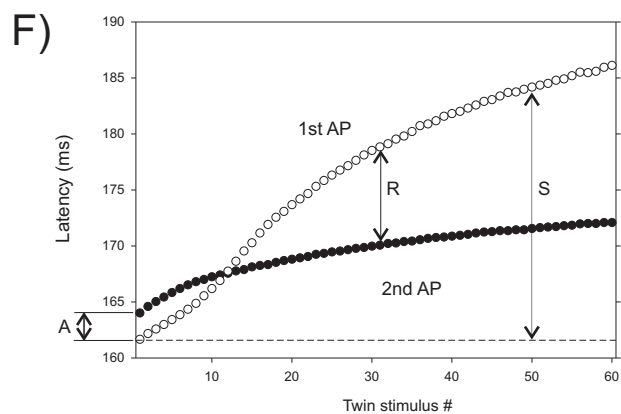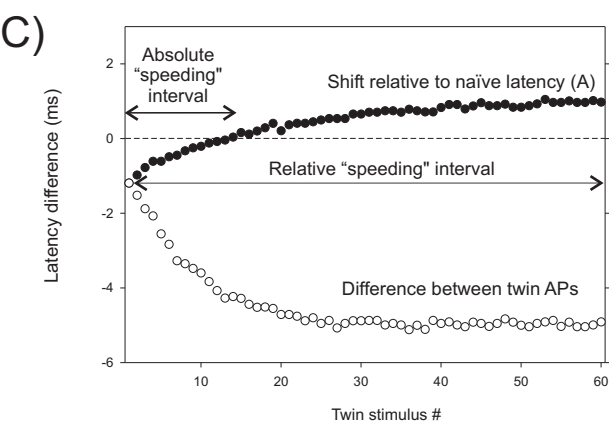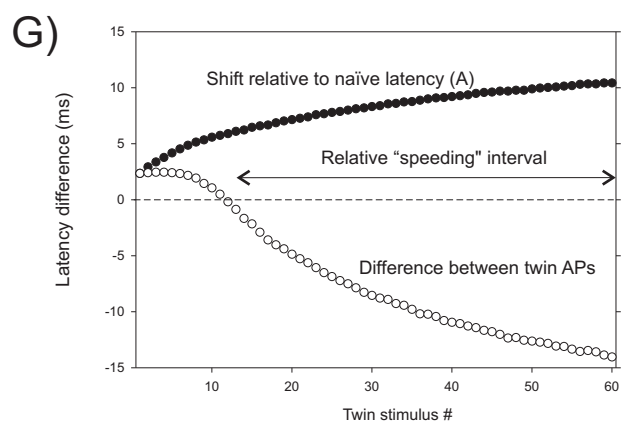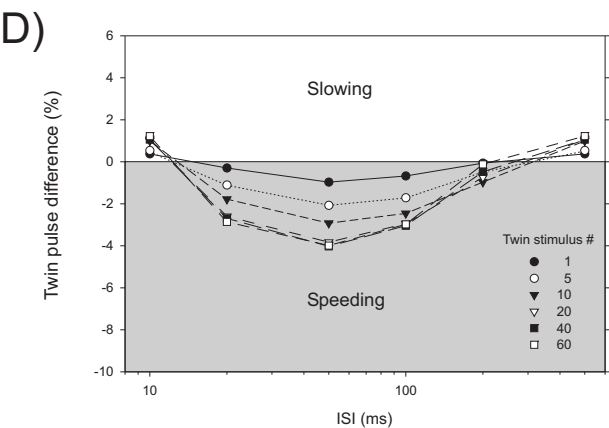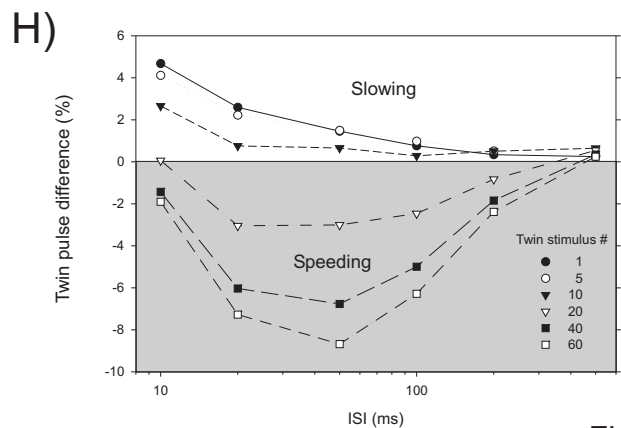

Fig. S2

Supplement: Figure S2 — Presumed sympathetic fiber shows absolute and relative speeding to 60 twin pulses. A recording from a C fiber is schematically summarized in Fig. 2A. For this presumed sympathetic fiber, transcutaneous electrical stimulation consisted of twin pulses delivered every 2 s with a stimulus interval between pulses of a twin stimulus of 50 ms. In response to twin stimulus #1, the first action potential (AP) arrived at the recording electrode 123 ms after the delivery of the first pulse at the cutaneous receptive field (see left vertical dashed line). Since the stimulus interval was 50 ms, the 2nd AP was expected to arrive at the recording electrode at least 173 ms after the first pulse. In other words, the 2nd AP was expected to arrive at the recording electrode about 50 ms following the 1st AP (indicated by the right vertical dashed line in Fig. 2A). However, the 2nd AP arrived at the recording electrode 171.8 ms after the first pulse, corresponding to a latency of 121.8 ms from the second pulse. Thus, the conduction of the 2nd AP was 1.2 ms faster than the conduction of the “naïve” AP (defined here as the first AP in the stimulus train). This phenomenon represents absolute “speeding” of conduction by 1% (marked by “A” in the trace for twin stimulus #1). For the second twin stimulus, the latency of the 1st AP (i.e., AP #3 in train) increased, as did the latency of the 2nd AP (AP #4). However, the latency of AP#4 was still smaller than the latency of the naive AP, indicating that absolute speeding still occurred. The relative latency of the 1st AP (S in figure) and the 2nd AP continued to increase during the course of the stimulation. At the end of the stimulus train (i.e., twin stim #60), the latency of the 1st AP (i.e., AP# 119) had increased to 129 ms (corresponding to a slowing of 4.9% relative to the naïve AP), whereas the latency of the 2nd AP (i.e., AP#120) had only increased to 124 ms. The latency of the 2nd AP was still shorter than the latency of the 1st AP, but [file pone.0009076.s002.pdf]

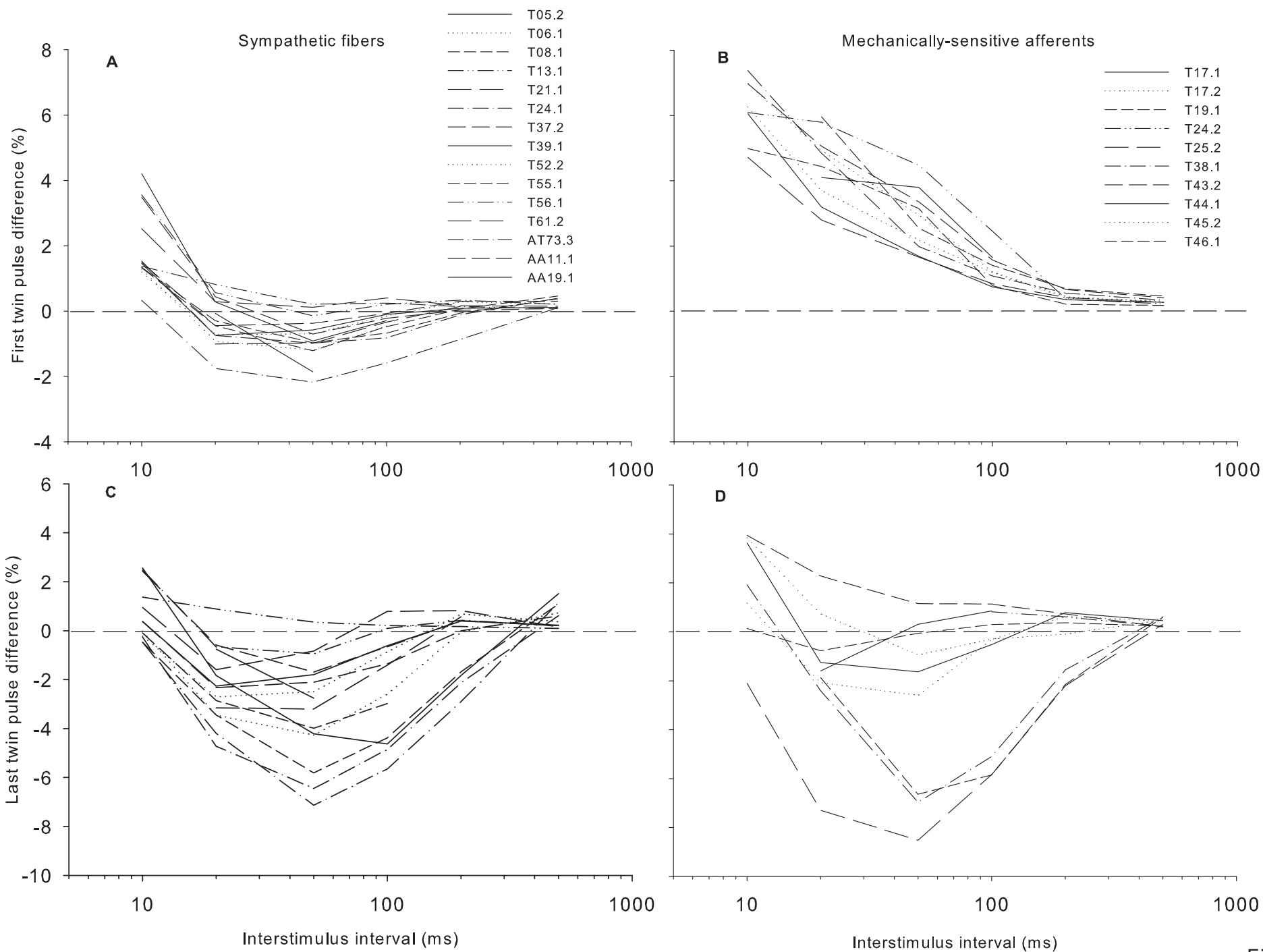

Fig. S3

Supplement: Figure S3 — Twin pulse data from mechanically-sensitive afferents and presumed sympathetic efferents. Fifteen presumed sympathetic fibers and 10 mechanically-sensitive afferents were studied with 2 or more different stimulus intervals using the repeated twin pulse paradigm. The twin pulse difference for the first twin pulse (A and B) and the last twin pulse (C and D) are plotted for these presumed sympathetic fibers (A and C) and mechanically-sensitive afferents (B and D). Each line corresponds to a different fiber. (0.07 MB PDF) [file pone.0009076.s003.pdf]

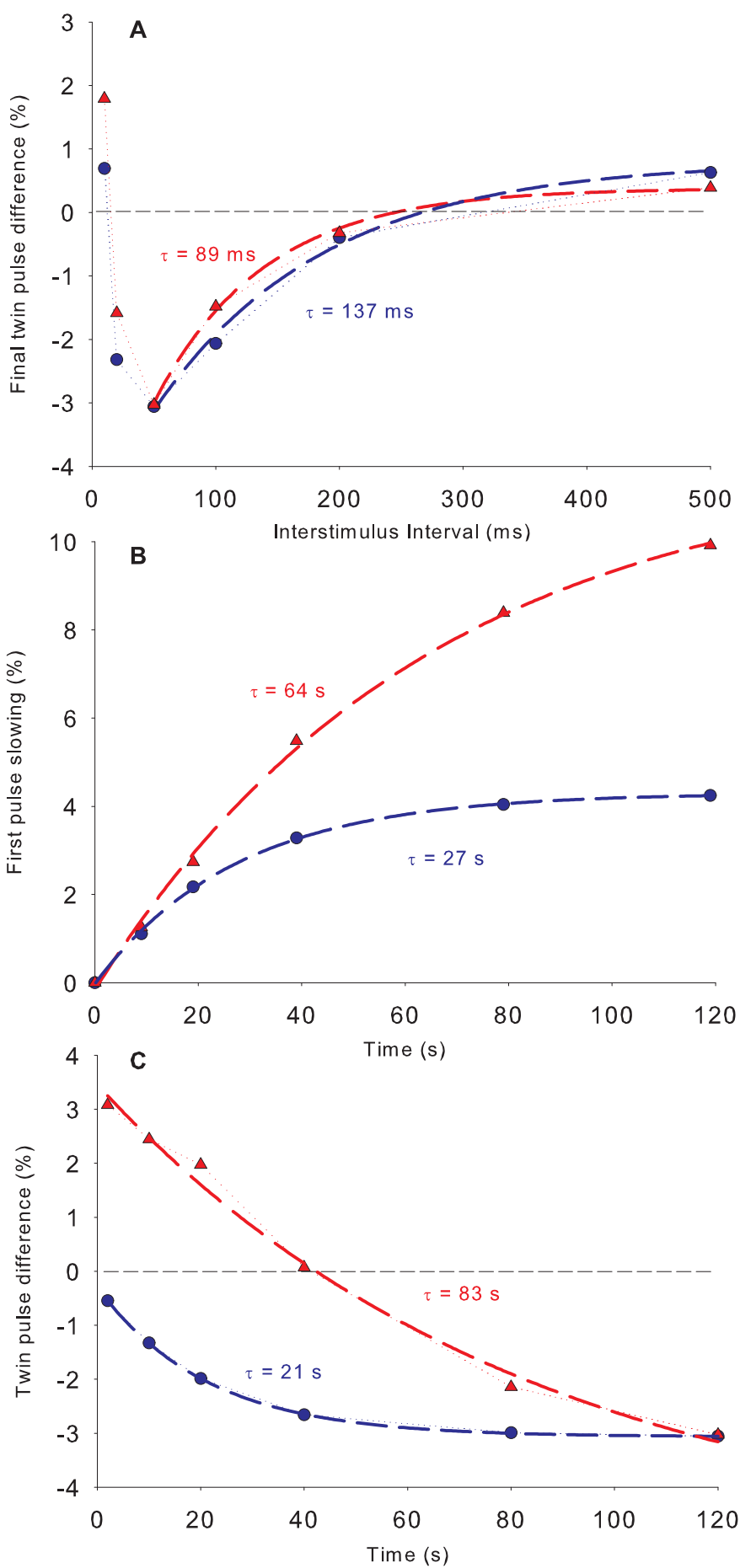

Fig. S4

Supplement: Figure S4 — Time constants. A. Recovery from twin pulse speeding. The average twin pulse difference for the last twin pulse is plotted as a function of stimulus interval for the mechanically-sensitive afferents (red triangles) and the presumed sympathetic fibers (blue circles). Exponential fits to the data over the interval from 50 to 500 ms are indicated by the dashed curves. The recovery time constant for the mechanically-sensitive afferents (89 ms) was shorter than for the presumed sympathetic fibers (137 ms). B. Activity-dependent slowing during the repeated twin pulse stimulation. The relative increase in latency of the first action potential in the twin pulse is plotted as a function of time during the twin pulse paradigm that lasted for 120 s for the mechanically-sensitive afferents (red triangles) and the presumed sympathetic fibers (circles). Exponential fits to the data are indicated by the dashed curves. Most of the activity-dependent slowing of the sympathetic fibers occurred in the first 20 pulses (time constant = 27 s). The activity-dependent slowing in the mechanically-sensitive afferents continued to increase throughout the paradigm (time constant = 64 s). The activity-dependent slowing of the first action potential was not dependent on the ISI of the twin pulse. C. Twin-pulse speeding during the repeated twin-pulse paradigm. The twin-pulse difference is plotted as a function of time during the twin-pulse paradigm. Average data for the 50 ms stimulus interval are used. For the presumed sympathetic fibers (blue circles), the twin-pulse difference starts negative (i.e., speeding); the magnitude of the twin-pulse difference increases for the first 20 pulses and then reaches a plateau (time constant = 21 s). For the mechanically-sensitive afferents (red triangles), the twin-pulse difference starts positive (i.e., twin-pulse slowing); the twin-pulse difference decreases throughout the paradigm reaching a negative value (i.e., speeding) comparable to the sympathetic f [file pone.0009076.s004.pdf]

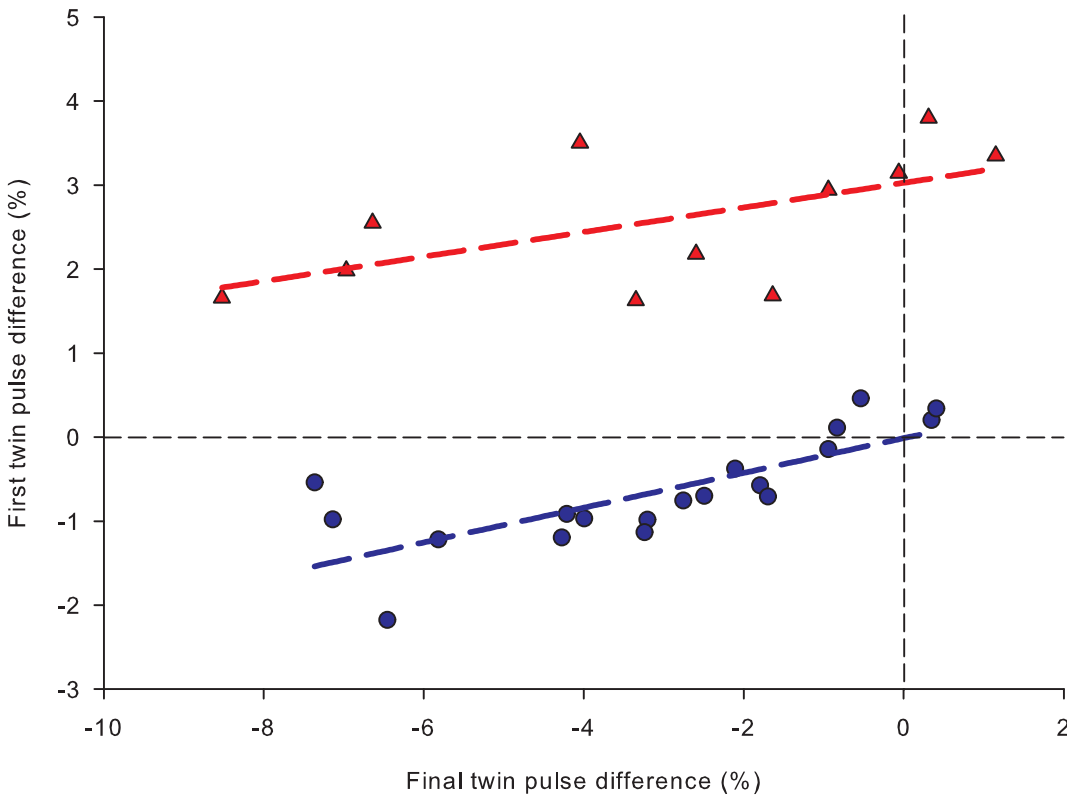

Fig. S5

Supplement: Figure S5 — First twin pulse latency correlates with last twin pulse latency. This scatter plot of first twin pulse latency versus last twin pulse latency (i.e, to the 60th twin pulse in the train) reveals a correlation for the sympathetic fibers (circles) (R2 = 0.59, p<0.001) and the afferent fibers (triangles) (R2 = 0.34, p = 0.058). Thus, sympathetic fibers that exhibited more initial twin pulse speeding developed more twin pulse speeding during the repeated stimulation than fibers with little initial twin pulse speeding. (0.03 MB PDF) [file pone.0009076.s005.pdf]
