# Supplementary material for: Conduction Properties Distinguish Unmyelinated Sympathetic Efferent Fibers and Unmyelinated Primary Afferent Fibers in the Monkey
Source: PLoS One. 2010 Feb 5;5(2):e9076. doi: 10.1371/journal.pone.0009076 (PMC2816714; doi:10.1371/journal.pone.0009076)
Supplement: Text S1 — Supplemental material. (0.05 MB DOC) [file pone.0009076.s006.doc]

***Supplemental material***

**Additional methods**

*Receptive field mapping.* The mechanical receptive field was mapped with suprathreshold von Frey hairs. For mechanically-insensitive fibers, an electrical receptive field was mapped with a hand-held saline-soaked cotton swab electrode in a manner described previously [1][2]. Briefly, a saline-soaked cotton swab was used to apply transcutaneous stimuli to the skin (with a needle inserted distally as the return electrode). The latency of the action potential at threshold increased gradually as the cotton swab was moved distally along the course of the nerve. At the electrical receptive field, the electrical threshold decreased dramatically and the latency increased. The electrical receptive field was defined as that area on the skin where discrete, stepped decreases in latency were observed as the stimulus intensity increased (e.g., see Fig. S1). Since only one action potential is recorded for each stimulus and the same action potential waveform is observed throughout the stimulation, these steps do not reflect recruitment of additional units, but correspond to different sites for action potential initiation within the terminal arbor. Superficial sites with long conduction latencies are activated at lower intensities, and deeper sites that are more distant from the stimulation electrode, are activated at higher stimulus intensities. The number of discrete steps and the size of the steps varied with position within the receptive field. Two or three spots were identified for subsequent investigation. To simplify the analysis, we chose to investigate spots where the number of steps was relatively small and the shift in latency for each adjacent step was relatively large.

*Chemical stimulation of mechanically-insensitive fibers.* Capsaicin (Sigma Aldrich, St. Louis, MO) was prepared in a vehicle of Tween 80 and saline as described previously [3] to achieve a dose of 10 g in a 10 l volume. Histamine dihydrochloride (Sigma-Aldrich, St. Louis, MO) was prepared in a saline vehicle at a dose of 10 g/10 l. The 10 l injections were made into the middle of the electrical receptive field and recordings were made for 10 min. The response to vehicle was always assessed before the response to the active substance. Units were classified as “responders” to a capsaicin or histamine if at least 10 action potentials (APs) were observed within 5 min after stimulus application, and the number of APs was at least twice that in response to the inactive substance.

**Voltage- latency curve for an afferent obtained by electrical stimulation at the receptive field using the well electrode** **(Figure S1).**

Before starting with the electrical stimulation protocols at the cutaneous terminals, electrical stimuli of constant duration (1 ms) but of increasing intensity were applied every 4 s. Threshold for electrical activation and the latency at this stimulus intensity were measured. Intensity was increased until a step decrease in conduction latency was observed. The stimulus intensity necessary to produce this step and the resulting new latency were noted. Intensity was increased to the upper limit of the Grass constant-voltage stimulator (150 V) or the Digitimer Constant Current stimulator (100mA). Stimulus intensities and conduction latencies were used to generate voltage-latency curves similar to the example shown in Fig. S1. At this stimulation site, the electrical threshold for activation was 60V, and the resulting conduction latency was about 125 ms. Up to an intensity of 85V, the conduction latency was stable, but at an intensity above 85 V, the latency stepped down to about 123 ms. Another latency step was observed with stimulus intensities above 99V at which the latency decreased to about 115 ms. No additional decrease in conduction latency was observed up to an intensity of 150V. These different latency levels correspond to discrete action potential initiation sites within the cutaneous arbor of the afferent. The purpose of these voltage-latency curves was to identify a wide stimulus intensity window over which the latency of the unit under study was stable to insure that the AP initiation site was fixed. For the subsequent electrical test protocols, stimulus intensity was usually set half way between the upper and lower limits of such a stable window. For this particular fiber, the stimulator was set at 125 V.

**Presumed sympathetic fiber shows absolute and relative speeding to 60 twin pulses (Figure S2, A-D)**

A recording from a C fiber is schematically summarized in Fig. 2A. For this presumed sympathetic fiber, transcutaneous electrical stimulation consisted of twin pulses delivered every 2 s with a stimulus interval between pulses of a twin stimulus of 50 ms. In response to twin stimulus #1, the first action potential (AP) arrived at the recording electrode 123 ms after the delivery of the first pulse at the cutaneous receptive field (see left vertical dashed line). Since the stimulus interval was 50 ms, the 2^nd^ AP was expected to arrive at the recording electrode at least 173 ms after the first pulse. In other words, the 2^nd^ AP was expected to arrive at the recording electrode about 50 ms following the 1^st^ AP (indicated by the right vertical dashed line in Fig. 2A). However, the 2^nd^ AP arrived at the recording electrode 171.8 ms after the first pulse, corresponding to a latency of 121.8 ms from the second pulse. Thus, the conduction of the 2^nd^ AP was 1.2 ms faster than the conduction of the “naive” AP (defined here as the first AP in the stimulus train). This phenomenon represents absolute “speeding” of conduction by 1% (marked by “A” in the trace for twin stimulus #1).

For the second twin stimulus, the latency of the 1^st^ AP (i.e., AP #3 in train) increased, as did the latency of the 2^nd^ AP (AP #4). However, the latency of AP#4 was still smaller than the latency of the naive AP, indicating that absolute speeding still occurred.

The relative latency of the 1^st^ AP (S in figure) and the 2^nd^ AP continued to increase during the course of the stimulation. At the end of the stimulus train (i.e., twin stim #60), the latency of the 1^st^ AP (i.e., AP# 119) had increased to 129 ms (corresponding to a slowing of 4.9% relative to the naïve AP), whereas the latency of the 2^nd^ AP (i.e., AP#120) had only increased to 124 ms. The latency of the 2^nd^ AP was still shorter than the latency of the 1^st^ AP, but it was now longer than the latency of the naive AP. Thus, the 2^nd^ AP no longer showed absolute speeding, but it did show relative speeding with respect to the 1^st^ AP (marked by “R” in the trace for twin pulse #60).

The latencies of the two APs are plotted as a function of twin stimulus number in Fig. S2B. The latency of the naive AP (i.e., AP #1) is indicated by the horizontal line in the graph. The latency of the 2^nd^ AP was shorter than the latency of the naive AP up to twin stimulus #14, indicating the presence of absolute speeding (see arrow labeled “A”). Throughout the stimulus train, the latency of the 2^nd^ AP was smaller than the latency of the 1^st^ AP, indicating the presence of relative speeding (see arrow marked “R”). The relative latency of the 1^st^ AP (S) increases throughout the protocol indicating activity-dependent slowing.

In Fig. S2C, the difference between the latency of the 2^nd^ AP and the naive AP is plotted as a function of the twin stimulus number (filled symbols). For the first 14 pairs of twin pulses, this difference was negative indicating absolute speeding. For the remaining twin stimuli, this difference became positive as the latency of the 2^nd^ AP in a given twin stimulus became larger than the latency of the naive AP. Fig. S2C also shows the latency difference between the 2^nd^ AP and the 1^st^ AP for every twin stimulus ( i.e., the latency difference between twin APs, open symbols). Since the 2^nd^ AP of a given twin stimulus always had a smaller latency than the preceding 1^st^ AP of the same twin stimulus, the latency difference between twin APs was always negative, indicating the presence of relative speeding throughout the stimulation. As can be seen, this difference became more negative during the first 25 pairs of twin stimuli after which it reached a plateau. As shown in Fig. S2B, this plateau is due to the fact that the conduction latencies of both action potentials had reached a plateau by the 25^th^ pair of twin stimuli, and that they increased only minimally throughout the rest of the train.

To analyze the latency data across different fibers, the latency difference between twin APs of a given twin stimulus was normalized by dividing by the latency to the naive AP of that stimulus train and is referred to as the “twin pulse difference.” In Fig. S2D, the twin pulse difference data for this fiber are plotted as a function of stimulus intervals (10, 20, 50, 100, 200 and 500 ms). Each curve corresponds to data collected at a given twin stimulus number during the different trains (i.e., twin stimulus number 1, 10, 20, 40, 60). In this fiber, the stimulus intervals of 10 and 500 ms resulted only in positive latency differences, indicating that the 2^nd^ AP was always conducted slower than the 1^st^ AP of the twin stimulus. Moreover, the positive difference did not change markedly from the first to the 60^th^ twin stimulus. In contrast, the latency differences between twin APs collected at the stimulus intervals of 20 - 200 ms were all negative. Specifically, this difference was negative with the first twin stimulus, i.e. absolute speeding was observed at these intra twin pulse intervals. Furthermore, the latency difference between twin APs became more negative for the first 20 twin stimuli, after which the difference reached a plateau and no further increase was observed.

**C-fiber mechanosensitive nociceptive afferent shows only relative speeding (Fig. S2, E-H).**

The right hand column of Fig. S2 shows a recording in a typical C-mechanosensitive afferent (C-MSA). The interval between the twin pulses was 50 ms, and twin stimuli were applied every 2 s. As shown in Fig. S2E, the second AP in response to twin stimulus #1 was conducted slower than the naive AP. With ongoing stimulation, the latencies of both APs increased, i.e. the conduction for both action potentials slowed. However, at the end of the stimulus train, the second AP (AP # 120) occurred less than 50 ms after the first AP (AP #119), indicating relative speeding of the second AP. When the latencies for the first and second action potential are plotted separately (see Fig. 2F), the development of relative speeding becomes more evident. For the first 11 twin stimuli, the latency of the second AP was greater than the latency of the naive AP which is marked by the horizontal line. In addition, the latency of the second AP was larger than the latency of its preceding AP. At twin stimulus # 12 the latencies from both APs were almost identical. After the 12^th^ twin stimulus, the latency of the first AP became larger than the corresponding second AP, i.e. relative speeding (R) developed. At the end of the stimulus train, the latency of the first AP had increased by a total of 20 ms to 186 ms, whereas the latency of the second AP had only increased by 9 ms. Fig. 2G shows the latency differences between the second AP of a twin stimulus and the naive AP (filled symbols). Since the second AP was always conducted slower than the naive AP, this difference was always positive. This is in contrast to the latency difference between the second and its preceding AP of a given twin stimulus which is represented by the open symbols in Fig. 2G. In the beginning of the stimulus train, this difference was positive, but with ongoing stimulation, it became smaller. With twin stimulus #12, this difference became negative, again illustrating the development of relative speeding. Relative speeding increased over the stimulus train and at the end of the stimulation the second AP was conducted 14 ms faster than the preceding AP.

Fig. 2H summarizes the normalized data that were collected for the different intra twin pulse intervals used. As can be seen, the latency difference between the twin APs of the twin stimulus #1 was always positive regardless of the stimulus interval, i.e. slowing of the second AP was always observed. Between twin stimuli 10 and 20 the difference became negative indicating the development of relative speeding. For all stimulus intervals between 20- 200 ms, relative speeding increased with the number of twin stimuli applied. No major changes in conduction latency were observed when the stimulus interval was set at 500 ms.

**Twin pulse data from mechanically-sensitive afferents and presumed sympathetic efferents (Figure S3).**

Fifteen presumed sympathetic fibers and 10 mechanically-sensitive afferents were studied with 2 or more different stimulus intervals using the repeated twin pulse paradigm. The twin pulse difference for the first twin pulse (A and B) and the last twin pulse (C and D) are plotted in Fig. S3 for these presumed sympathetic fibers (A and C) and mechanically-sensitive afferents (B and D). Each line corresponds to a different fiber.

**Time constants (Figure S4)**

**A. Recovery from twin pulse speeding.** The average twin pulse difference for the last twin pulse is plotted as a function of stimulus interval for the mechanically-sensitive afferents (red triangles) and the presumed sympathetic fibers (blue circles). Exponential fits to the data over the interval from 50 to 500 ms are indicated by the dashed curves. The recovery time constant for the mechanically-sensitive afferents (89 ms) was shorter than for the presumed sympathetic fibers (137 ms).

**B. Activity-dependent slowing during the repeated twin pulse stimulation.** The relative increase in latency of the first action potential in the twin pulse is plotted as a function of time during the twin pulse paradigm that lasted for 120 s for the mechanically-sensitive afferents (red triangles) and the presumed sympathetic fibers (circles). Exponential fits to the data are indicated by the dashed curves. Most of the activity-dependent slowing of the sympathetic fibers occurred in the first 20 pulses (time constant = 27 s). The activity-dependent slowing in the mechanically-sensitive afferents continued to increase throughout the paradigm (time constant = 64 s). The activity-dependent slowing of the first action potential was not dependent on the ISI of the twin pulse.

**C. Twin-pulse speeding during the repeated twin-pulse paradigm.**  The twin-pulse difference is plotted as a function of time during the twin-pulse paradigm. Average data for the 50 ms stimulus interval are used. For the presumed sympathetic fibers (blue circles), the twin-pulse difference starts negative (i.e., speeding); the magnitude of the twin-pulse difference increases for the first 20 pulses and then reaches a plateau (time constant = 21 s). For the mechanically-sensitive afferents (red triangles), the twin-pulse difference starts positive (i.e., twin-pulse slowing); the twin-pulse difference decreases throughout the paradigm reaching a negative value (i.e., speeding) comparable to the sympathetic fibers by the end of the paradigm (time constant = 83 s).

**First twin pulse latency correlates with last twin pulse latency (Figure S5)**

This scatter plot of first twin pulse latency versus last twin pulse latency (i.e, to the 60th twin pulse in the train) reveals a correlation for the sympathetic fibers (circles) (R^2^ = 0.59, p < 0.001) and the afferent fibers (triangles) (R^2^ = 0.34, p = 0.058). Thus, sympathetic fibers that exhibited more initial twin pulse speeding developed more twin pulse speeding during the repeated stimulation than fibers with little initial twin pulse speeding.

Reference List

1. Meyer RA, Davis KD, Cohen RH, Treede R-D, Campbell JN (1991) Mechanically insensitive afferents (MIAs) in cutaneous nerves of monkey. Brain Res 561: 252-261.

2. Peng YB, Ringkamp M, Campbell JN, Meyer RA (1999) Electrophysiological assessment of the cutaneous arborization of A-delta-fiber nociceptors. J Neurophysiol 82: 1164-1177.

3. LaMotte RH, Shain CN, Simone DA, Tsai E-FP (1991) Neurogenic hyperalgesia: Psychophysical studies of underlying mechanisms. J Neurophysiol 66: 190-211.
